# Serum antibody fingerprinting of SARS-CoV-2 variants in infected and vaccinated subjects by label-free microarray biosensor

**DOI:** 10.3389/fimmu.2024.1323406

**Published:** 2024-02-27

**Authors:** Thomas Carzaniga, Luca Casiraghi, Giovanni Nava, Giuliano Zanchetta, Tommaso Inzani, Marcella Chiari, Valentina Bollati, Sara Epis, Claudio Bandi, Alessia Lai, Gianguglielmo Zehender, Tommaso Bellini, Marco Buscaglia

**Affiliations:** ^1^ Dipartimento di Biotecnologie Mediche e Medicina Traslazionale, Università degli Studi di Milano, Milan, Italy; ^2^ Istituto di Scienze e Tecnologie Chimiche “Giulio Natta”, National Research Council of Italy (SCITEC-CNR), Milano, Italy; ^3^ Dipartimento di Scienze Cliniche e di Comunità, Università degli Studi di Milano, Milano, Italy; ^4^ Dipartimento di Bioscienze and Pediatric Clinical Research Center (CRC) ‘Fondazione Romeo ed Enrica Invernizzi’, Università degli Studi di Milano, Milano, Italy; ^5^ Dipartimento di Scienze Biomediche e Cliniche, Università degli Studi di Milano, Milano, Italy

**Keywords:** label-free biosensor, reflective phantom interface, antibody repertoire, rapid detection, wash-free assay, serological assay, immunoglobulins, IgA

## Abstract

Both viral infection and vaccination affect the antibody repertoire of a person. Here, we demonstrate that the analysis of serum antibodies generates information not only on the virus type that caused the infection but also on the specific virus variant. We developed a rapid multiplex assay providing a fingerprint of serum antibodies against five different SARS-CoV-2 variants based on a microarray of virus antigens immobilized on the surface of a label-free reflectometric biosensor. We analyzed serum from the plasma of convalescent subjects and vaccinated volunteers and extracted individual antibody profiles of both total immunoglobulin Ig and IgA fractions. We found that Ig level profiles were strongly correlated with the specific variant of infection or vaccination and that vaccinated subjects displayed a larger quantity of total Ig and a lower fraction of IgA relative to the population of convalescent unvaccinated subjects.

## 1 Introduction

The easy access to individual antibody repertoire, which results from a complex interplay of factors (1), would constitute an important achievement in providing epidemiological information, controlling disease outbreaks, and developing effective clinical therapeutics and vaccine strategies (2). During the COVID-19 pandemic, quantitative measurement of SARS-CoV-2 antibody titer enabled assessing variability in the immune response to infection, evaluating vaccine efficacy and potential for long-term immunity, and identifying donors for blood transfusion therapy (3–10). Large-scale antibody quantification and characterization are commonly accomplished using enzyme-linked immunosorbent assay (ELISA) in laboratory facilities and lateral flow assay (LFA) as the rapid serological test at the point of care (POC). Notably, both ELISA and LFA do not allow parallel quantification of distinct antibodies and are thus not suitable for the fingerprinting of antibody repertoire (11–13).

Multiplexed antigen assay platforms represent a key development for the accurate identification of antibody repertoires (14). A high-throughput approach is offered by peptide microarrays, which enable identifying immunoreactive epitopes from the blood of individuals with different histories of exposure to infective agents (15–19). The relevance of this approach in serodiagnostics is, however, still to be confirmed. In the context of SARS-CoV-2, a few multiplexed antigen assay platforms have been proposed, which include fluorescence protein microarray (20, 21), as well as bead-based approaches (22). Despite the validity of these methodologies, the COVID-19 pandemic experience has highlighted the critical need for affordable new assay formats that offer highly sensitive, quantitative, multiplexed, and rapid immune protection profiling.

Here, we show that it is possible to discriminate antibody repertoires in serum up to the resolution of a single SARS-CoV-2 virus variant with a simple yet sensitive and quantitative assay based on label-free readout of an antigen microarray, without additional markers to provide the signal (e.g., colorimetric, fluorescent, and chemiluminescent). With the same multiplex assay, it is also possible to discriminate between vaccinated and unvaccinated subjects through their total Ig profile and IgA amount. These results are based on a multi-spot biosensing technique, the reflective phantom interface (RPI) (23, 24), which enables real-time quantification of molecular binding. Overall, the proposed antibody fingerprinting method paves the way to POC characterization of antibody repertoire against specific panels of protein antigens for purposes of either individual diagnostic or population screening.

## 2 Methods

### 2.1 Serum samples, reagents, and materials

All receptor-binding domain (RBD) of SARS-CoV-2 spike proteins (WT-RBD, *α*-RBD, *γ*-RBD, *δ*-RBD, and *o*-RBD) obtained from HEK293 human embryonic kidney immortalized cell line were purchased from Sino Biological (Beijing, China). Nucleocapsid protein was obtained from InvivoGen (Toulouse, France; product code his-sars2-n). WT-LtRBD was expressed in the protozoa parasite *Leishmania tarentolae* and purified (Patent N. IT 10202100000416 25). Trimeric spike protein HexaPro was donated by Anton Schmitz and Günter Mayer (26, 27). Rabbit polyclonal antibody anti-human IgG was obtained Abcam (Cambridge, UK; product code ab7155). Goat polyclonal antibody anti-human IgA was obtained from Invitrogen (Rockford, IL, USA; product code SA5-10252). Wedge-like glass chips (F2 optical glass, Schott), with a 5° angle, a maximum thickness of 2 mm, and a size of 8 mm × 12 mm, were coated with SiO_2_ to form an anti-reflection layer of 80 nm. After ozone cleaning, the chips were dip-coated with a copolymer of dimethylacrylamide (DMA), *N*-acryloxysuccinimide (NAS), and 3-(trimethoxysilyl)propyl methacrylate (MAPS)–copoly(DMA–NAS–MAPS) called MCP2 purchased from Lucidant Polymers Inc. (Sunnyvale, CA, USA; 28). All the buffers and reagents were purchased from Sigma-Aldrich (St. Louis, MO, USA) and prepared using Milli-Q pure water. Plasma samples were obtained from healthy volunteer donors and patients at the Sacco Hospital in Milan.

### 2.2 Preparation of RPI antigen microarray cartridge

Antigen proteins and control antibodies were covalently immobilized on the surface of RPI sensing chips in spots with 150–200-μm diameter. Droplets of spotting buffer (phosphate-buffered saline (PBS) 1×, pH 7.4, and trehalose 50 mM) containing probe proteins at concentrations of 1 mg/mL were deposited on the chip surface by an automated, non-contact dispensing system (sciFLEXARRAYER S3; Scienion AG, Berlin, Germany). After overnight incubation, the chip surface was rinsed with blocking buffer (Tris-HCl, pH 8, 10 mM, NaCl 150 mM, and ethanolamine 50 mM) and distilled water and then dried. The disposable sensor cartridges were prepared by gluing the glass chips on the inner wall of 1-cm plastic cuvettes. The cartridges were stored at 4°C before use.

### 2.3 Label-free microarray measurements

The measurements were performed using the RPI apparatus described in Salina et al. (24), comprising LED illumination with wavelength centered at 455 nm and acquisition of images of reflected light by a CCD camera (Stingray F-145C, Allied Vision, Stadtroda, Germany). The sensor cartridges were filled with 1.3 mL of measuring buffer (PBS 1×, pH 7.4, SDS 0.02% and Sodium azide 0.02%) and adding 13 μL of plasma was addedd with a micropipette. The cartridges were kept at 23°C during the measurement through a thermalized holder, and rapid mixing of the solution was provided by a magnetic stirring bar rotating at 30 Hz. Time sequences of RPI images were acquired at 12 fps, and 60 consecutive images were averaged to provide a final set of images corresponding to 5 seconds of total acquisition time per image. After a time *t*_1_ = 1 hour of acquisition, the cuvette was emptied and filled again with measuring buffer. Then, anti-IgA antibodies were added in solution to a final concentration of 50 nM, and a second set of images was acquired for a time *t*_2_ = 1 hour.

### 2.4 Data analysis

Time sequences of RPI images of the spotted surface were analyzed using a custom MATLAB program (The MathWorks, Natick, MA, USA) to obtain the brightness of each spot as a function of time *t* and converted into the total mass surface density of molecules *σ*(*t*). The conversion of the brightness of the RPI image pixels *u_s_* (*t*) into surface density is performed according to the following:


(1) $$\sigma (t)\;=\sigma^{*}\sqrt{(u_{s}(t)/u_{0})-1}-\delta \sigma$$

where *σ* ^∗^, *u*_0_, and *δσ* are obtained as described in Salina et al. (24) from the physical parameters of the RPI sensor, the refractive index of the solution, and the density and refractive index of a compact layer of biomolecules on the surface. The mass surface density of antibody binding the surface-immobilized antigens was obtained as Δ*σ*(*t*) = *σ*(*t*) − *σ*_0_, where *σ*_0_ is the surface density measured before the addition of plasma sample. The analysis of the binding curves was performed on Δ*σ*(*t*) traces obtained by averaging at least five spots with identical composition. Each binding curve was fitted with the exponential growth function:


(2) $$\Delta \sigma (t)\;=\;\Delta \sigma_{eq}(1-e^{-kt})$$

where Δ*σ_eq_* is the asymptotic amplitude and *k* is the observed binding rate. The growth unit was obtained from the derivative of **Equation 2** as Equation 3 or Equation 4. *RGU* was obtained as the ratio between the *GU_Ig_* of each variant RBD and that of WT-RBD.

## 3 Results

### 3.1 Principle of antigen microarray biosensor

**Figure 1:**
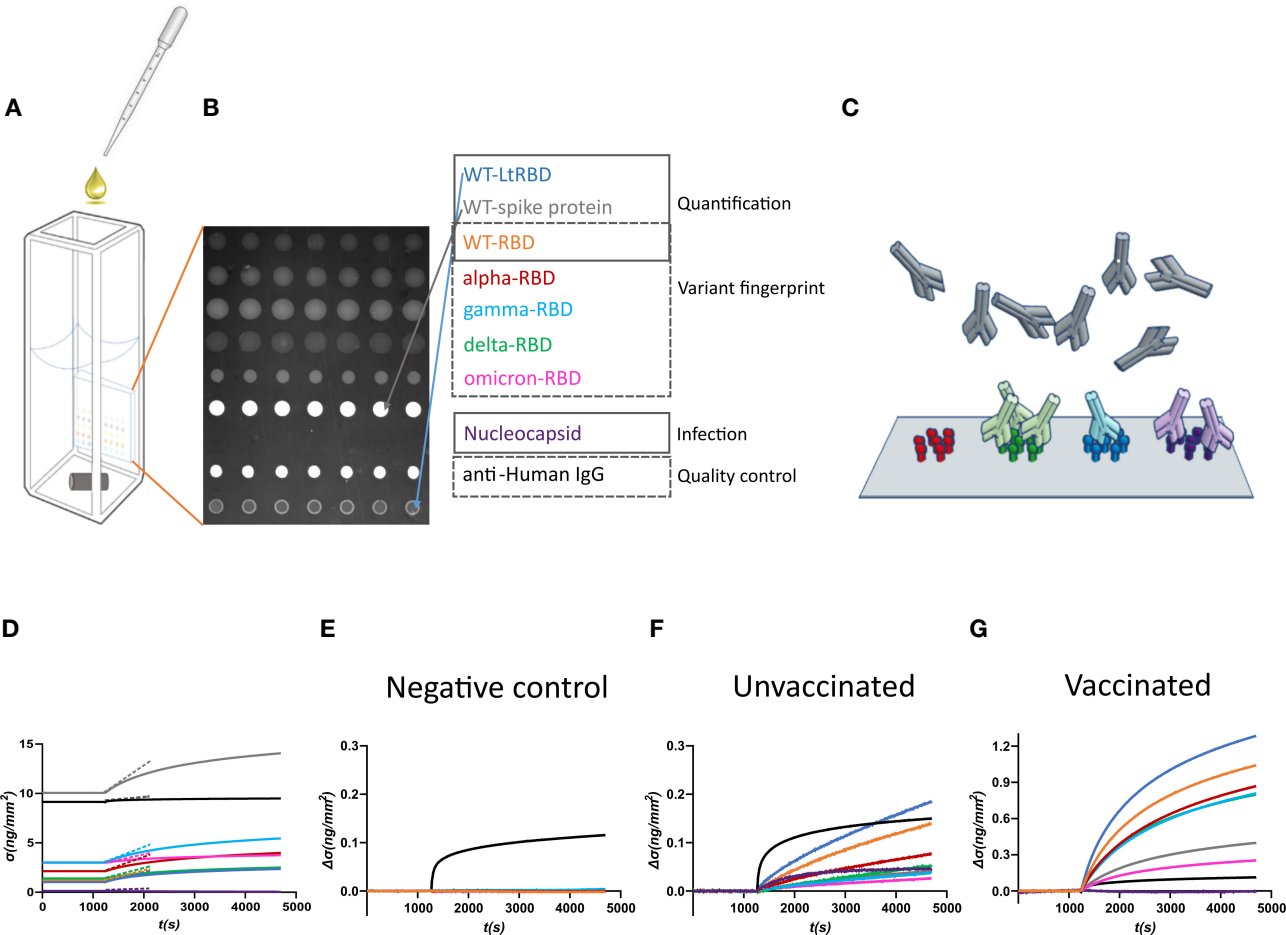
Design of the label-free microarray for anti-SARS-CoV-2 antibody fingerprinting. **(A)** Schematic of the plastic cartridge with the reflective phantom interface (RPI) sensor glued on an inner wall. Plasma samples were added to the measuring buffer using a pipette, and mixing was performed using a rotating stirring bar. **(B)** RPI image of the sensing surface spotted with SARS-CoV-2 antigens and control proteins as indicated in the legend. **(C)** Cartoon of spotted sensing surface representing the binding of serum antibodies on the corresponding immobilized antigens. **(D)** Example of raw data of *σ*(*t*) measured before (baseline, *σ*(*t*) = *σ*_0_) and after the addition of 1:100 dilution of plasma sample. The dashed lines represent the initial slope of the binding curves. **(E)** Surface density Δ*σ*(*t*) = *σ*(*t*) − *σ*_0_ measured upon addition of plasma sample collected before the COVID-19 pandemic. Only the spot of anti-IgG antibody provides a signal, whereas no binding was observed on antigen spots. **(F)** Surface density Δ*σ*(*t*) measured upon addition of plasma sample of an unvaccinated subject previously infected by SARS-CoV-2. All antigen spots provide a positive signal but with different amplitudes. **(G)** Surface density Δ*σ*(*t*) measured upon addition of plasma sample of a vaccinated subject. The signal is generally larger than that obtained for convalescent subjects.

Antibody fingerprints were obtained from convalescent subjects, either vaccinated or unvaccinated, exposed to different virus variants. Plasma samples were inserted in the measuring cell (**Figure 1A**) hosting the RPI sensor surface (**Figure 1B**), which was prepared to immobilize different antigens in the form of a multi-spot microarray. The antigen panel was composed of the following: i–v) five types of recombinant RBD of the spike protein of SARS-CoV-2 expressed in human cells (HEK293), corresponding to WT (WT-RBD), alpha (*α*-RBD), gamma (*γ*-RBD), delta (*δ*-RBD), and omicron (*o*-RBD) variants; vi) full trimeric WT spike protein expressed in human cells; vii) a variant of WT-RBD expressed in *L. tarentolae* (25) (WT-LtRBD), added to evaluate the effect of different antigen glycosylation; viii) nucleocapsid protein; and ix) antibody anti-human IgG as a positive control.

The RPI biosensor substrate consists of a wedge-shaped glass slab coated with a thin film of SiO_2_ providing anti-reflective conditions in water and with a multi-functional copolymer of dimethylacrylamide (29). When observed in the back-reflection direction, spots appear as brighter disks because the bio-conjugated proteins and the antibodies, that in time accumulate on them (**Figure 1C**), provide an additional effective thickness relative to the optimized anti-reflective coating condition. Images of the spotted surface (**Figure 1B**) were acquired before and after the addition of plasma, and the brightness of each spot was converted into surface mass density *σ* (**Equation 1**) expressed in ng/mm^2^ (24). The value of *σ* reflects both the size and density of the total surface accumulation of antigens and antibodies.

**Figure 1D** shows an example of the assay response upon the addition of 1:100 dilution of human plasma from a vaccinated subject. *σ*_0_, the value of *σ* before the plasma addition, is larger in the spots of full spike protein and control IgG because of their larger molecular size. After the plasma addition, the molecular density on all spots increases. **Figures 1E–G** report three examples of different types of response of the increment Δ*σ*(*t*) = *σ*(*t*) − *σ*_0_ with time *t*, observed upon addition of pre-COVID-19 pandemic human plasma (**Figure 1E**), and of plasma from an unvaccinated convalescent subject (**Figure 1F**) or from a previously uninfected vaccinated subject (**Figure 1G**). Pre-pandemic plasma components show negligible non-specific binding; the only significant signal is due to the presence of immunoglobulins on the anti-human IgG spot. In contrast, Δ*σ*(*t*) increases for all the antigen spots in the other two cases. The differences in the response to the antigens between classes of subjects and within each subject enable pinpointing infection history-dependent anti-SARS-CoV-2 immunoglobulin repertoires.

### 3.2 Serum antibody fingerprint of subjects exposed to different SARS-CoV-2 variants

To quantify the relative amount of antibodies binding to the different antigens, we used the growth units *GU_Ig_* determined from the growth rate of *σ*(*t*) right after the plasma addition at *t* = *t*_0_ normalized by the initial surface density of the spot (**Equations 2** and 3). The parameter *GU_Ig_*, being based on the slope of the linear growth of *σ*(*t*) at short times (straight lines in **Figure 1D**), can be obtained with precision after a few minutes, much shorter than the time needed to estimate the asymptotic equilibrium value of the binding curve, although it provides equivalent information. We extracted the *GU_Ig_* from each antigen of each plasma sample, as well as the ratio *RGU* between the *GU_Ig_* of each variant RBD and that of WT-RBD, which we adopted as an internal reference to extract accurate antibody fingerprints.

**Figure 2:**
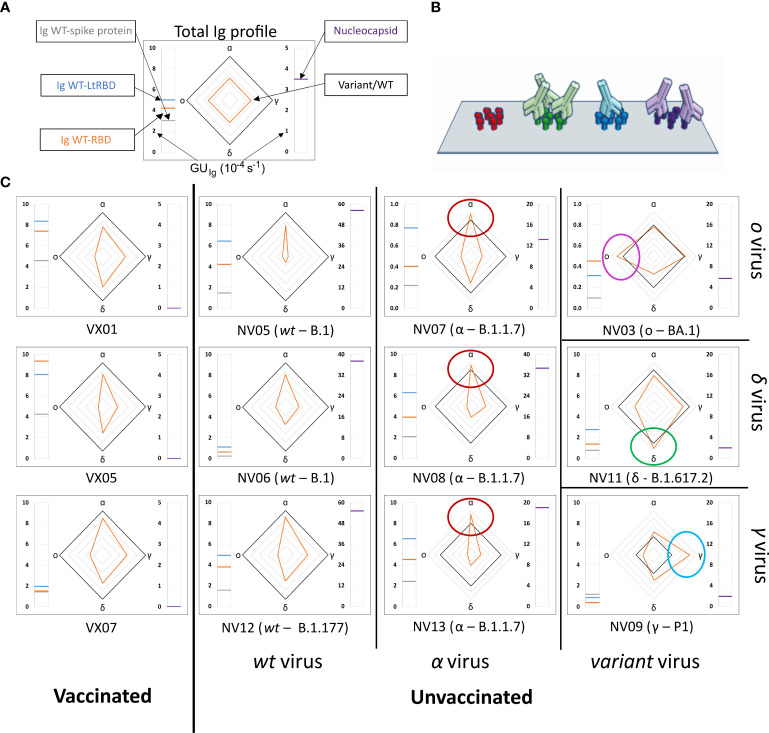
Immunoglobulins fingerprint against antigens of different SARS-CoV-2 variants. **(A)** Legend of the fingerprint diagram. The left-side meter reports the quantification of Ig in terms of *GU_Ig_* of three WT antigens, as indicated. The right-side meter reports the quantification of anti-nucleocapsid antibodies expressed as *GU_Ig_*. The radar chart reports the values of *RGU* for alpha, gamma, delta, and omicron RBD variants. The thick black contour line represents the number of antibodies binding to WT-RBD taken as reference, hence corresponding to *RGU* = 1. **(B)** Cartoon of the assay design: Ig antibodies bind the surface-immobilized antigens. **(C)** Selection of Ig fingerprints obtained for three samples of vaccinated subjects (left column) and nine samples of convalescent subjects infected with different variants of SARS-CoV-2: WT (second column from the left), alpha (third column from the left), gamma (right column, bottom), delta (right column, center), and omicron (right column, top).

We analyzed plasma samples from 27 subjects, of whom 14 were vaccinated and 13 were unvaccinated and convalescent. The samples were collected between 2 and 22 days after the onset of symptoms or between 9 and 103 days after vaccination. Molecular tests identified variants of infections as WT, alpha, gamma, delta, or omicron, whereas all vaccines were against WT (**Supplementary Tables 1, 2**). The results obtained from a selection of samples are shown in **Figure 2**, where each box corresponds to plasma from a different subject and is organized into three parts. The meter on the left-hand side reports the values of *GU_Ig_* relative to full spike protein (gray line), WT-RBD (orange line), and WT-LtRBD (blue line), while the one on the right side shows the value of *GU_Ig_* for the nucleocapsid. In the center, we display a radar chart (orange line) to express *RGU* for the alpha, gamma, delta, and omicron RBD variants. The black line square serves as *RGU* = 1 reference. The condition *RGU >* 1 (orange vertex outside the reference square, marked by colored circles) indicates an Ig amount larger than that of WT-RBD.

Our data show a large variability of the absolute amount of Ig against the SARS-CoV-2 antigens among individuals, in agreement with previous reports (9, 10, 30). An example is provided by the meter data (left-hand side of the boxes in **Figure 2**), which show the absolute amount of Ig against full spike WT protein and WT-RBD domains, markedly different from individual to individual. Despite this variability, a surprisingly stable pattern emerges when the ratios between Ig amounts, as expressed by RGU, are considered. This finding can be appreciated in the radar charts, where i) both vaccinated and unvaccinated subjects infected by the WT virus consistently display a smaller amount of Ig for the other variants (orange line always inside the black square, i.e., *RGU<* 1) with antibodies against alpha RBD always in the largest amount and those targeting omicron RBD in the smallest amount, and ii) unvaccinated subjects infected by SARS-CoV-2 variants display a pronounced response to the corresponding antigen, by which, for example, subjects infected by alpha variant display antibodies binding to *α*-RBD in larger amounts than to any other variants. This capacity to discriminate infection variants from the antibody response crucially relies on the multiplexing structure of our assay, enabling simple computation of response ratios.

Nucleocapsid-binding Ig cannot be detected in vaccinated individuals (right-hand side meter), as expected, whereas convalescent subjects showed a variable amount of these antibodies.

**Figure 3:**
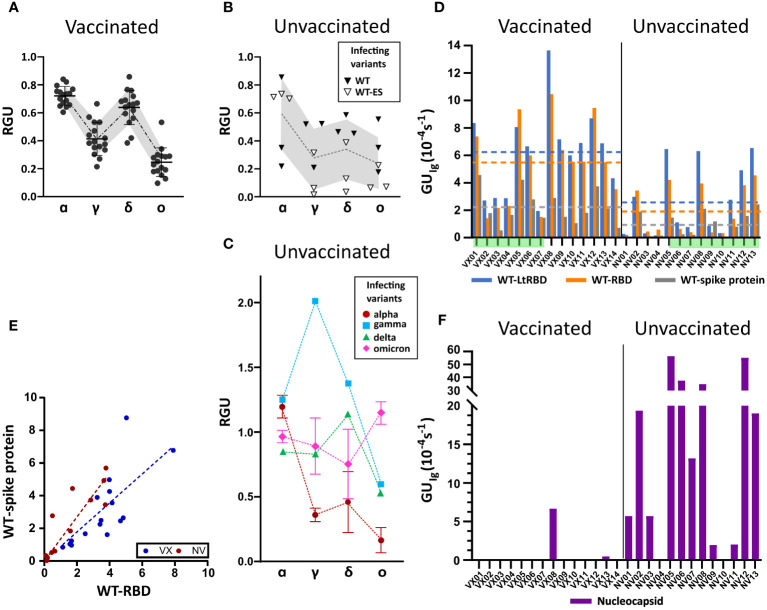
Ig fingerprint of vaccinated and unvaccinated convalescent subjects. **(A)** Summary of *RGU* measured for plasma samples of 14 vaccinated subjects. **(B)** Summary of *RGU* of six convalescent unvaccinated subjects infected with WT variant of strain B.1 (WT) or B.1.177 (WT-ES), as indicated in the legend. **A, B** The dotted lines connect the average values, and the borders of the shaded area connect the standard deviation values. **(C)** Summary of *RGU* of seven convalescent unvaccinated subjects infected with the variants indicated in the legend. Standard deviations are indicated for variants with multiple samples. **(D)** Relative quantification of total Ig anti-WT spike protein and RBD, WT-RBD, and WT-LtRBD, expressed as *GU_Ig_* for all the analyzed samples. The dashed lines represent the average values of *GU_Ig_* for all samples of vaccinated and unvaccinated subjects, with the color corresponding to different antigens as indicated in the legend. **(E)** Growth unit *GU_Ig_* of Ig on WT full spike protein and WT-RBD spots for vaccinated (blue) and unvaccinated (red) subjects. The lines represent linear fit to the data points with the corresponding color. **(F)** Relative quantification of anti-nucleocapsid Ig expressed as *GU_Ig_* for all the analyzed samples.

The analysis of the full set of plasma samples (**Supplementary Figures 1, 2**) confirms the general behavior exemplified in **Figure 2**. **Figures 3A–C** report the pattern of relative antibody efficiency expressed by *RGU* grouped as vaccinated, unvaccinated with past infection of WT, and unvaccinated with past infection of other variants, respectively.

All samples of vaccinated subjects (**Figure 3A**) display a similar pattern of response with *RGU<* 1. *A* similar hierarchy of binding signals is observed in samples from WT-infected convalescent subjects (**Figure 3B**), although in this case, data are much more spread in value (gray shading). In contrast, as anticipated in **Figure 2**, different patterns and a larger variability of *RGU* emerge for convalescent subjects infected with the other variants (**Figure 3C**). In this group, for each variant of infection, the strongest response is consistently against the corresponding RBD, with values of *RGU* larger than 1.

Overall, these results demonstrate that the label-free microarray composed of eight SARS-CoV-2 antigens can serve as antibody fingerprints accurate enough to discriminate between past infection and vaccination with different virus variants.

### 3.3 Comparison between vaccinated and unvaccinated subjects

Subjects vaccinated by WT antigen and convalescent unvaccinated subjects display *RGU* fingerprints with different features, as shown in **Figures 3A–C**. Further differences emerge from the analysis of the absolute quantification of Ig by *GU_Ig_*. **Figure 3D** shows that, on average, vaccinated subjects display larger quantities of specific antibodies against WT spike protein and RBD than unvaccinated subjects do. Data exhibit a large subject-to-subject variation coherent with the wide range of IgG concentrations, from 5 to 300 ng/mL, estimated by ELISA (**Supplementary Figure 3**). **Figure 3D** also indicates that the antibodies binding WT-LtRBD are more than those binding WT-RBD (blue *vs.* orange columns and lines) for both vaccinated and unvaccinated subjects. This is ascribed to slightly different glycosylation of WT-RBD expressed in human cell lines and WT-LtRBD. Even more significant is the difference in binding to the full spike protein (gray column and line), much weaker relative to WT-RBD for vaccinated subjects in comparison to convalescent ones. This difference is also shown in **Figure 3E**, where it appears that, for equal response to WT-RBD, unvaccinated convalescent subjects have on average a larger response to the full spike protein.

Finally, **Figure 3F** shows that anti-nucleocapsid Ig is only present in samples of convalescent subjects. This is expected since the SARS-CoV-2 nucleocapsid protein is not contained in vaccine formulation. Two samples of vaccinated subjects displayed anti-nucleocapsid Ig: VX08 was in prolonged contact with infected subjects after vaccination, and VX13 was presumably infected before vaccination since some symptoms were reported. As apparent from the vertical scales in **Figure 3F** vs. **3D**, the response to nucleocapsid is extremely variable among the subjects. Indeed, while a positive response to nucleocapsid is a clear indication of a previous infection, undetectable levels of anti-nucleocapsid antibodies are not necessarily an indication of the absence of previous infections, as in the case of samples NV04 and NV10, negative to nucleocapsid despite their past infection, as confirmed by molecular testing.

### 3.4 Serum immunoglobulin A fingerprint for SARS-CoV-2 exposure

**Figure 4:**
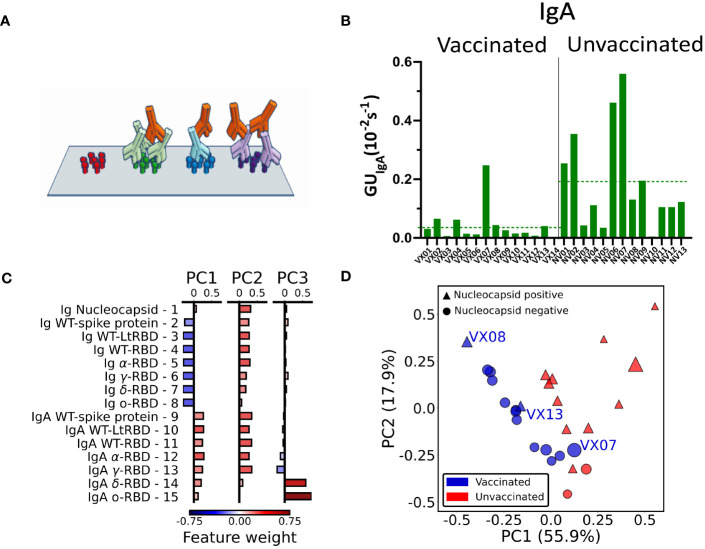
IgA fraction and principal component analysis of antibody fingerprint. **(A)** Cartoon of the IgA assay design: IgA antibodies (red) bind the Ig that previously bound the surface-immobilized antigen in the first step of the assay. **(B)** Fraction of IgA expressed as average value of *GU_IgA_* on all the RBD spots for all the analyzed samples. The dashed lines represent the average values for all samples of vaccinated and unvaccinated subjects. **(C)** Weight of each feature in the components PC1, PC2, and PC3 obtained by principal component analysis. **(D)** Plot of all the 27 plasma samples in the PC1–PC2 plane. The size of each data point is proportional to the value of the PC3 component; color and shape indicate if the subject was vaccinated (blue) or unvaccinated (red) and if the nucleocapsid detection was positive (triangle) or negative (circle).

The label-free assay can be enriched with the capability of discriminating between types of antibodies by measuring the binding of anti-antibodies to Ig already bound on the antigen spots after the first measuring step described above (**Figure 4A**). This was performed by replacing the plasma in the measuring cartridge with a buffer containing anti-IgA antibodies. In analogy to the quantification offered by *GU_Ig_*, we extracted from the data the slope of the initial linear growth of anti-IgA antibodies surface density *σ*(*t*), which we normalized to the surface density Δ*σ*(*t*_1_) of Ig at the end of the first measuring step (Equation 4). The resulting parameter (see Methods) represents the fraction of IgA in the Ig repertoire for a specific antigen.

Radar charts analogous to those in **Figure 2** are reported in **Supplementary Figures 4, 5**. Differently from the quantification of the total Ig, we found no evident correlation of *GU_IgA_* with the subject history. However, the overall fraction of IgA, estimated by the average *GU_IgA_* for all RBD variants, tends to be generally larger for the samples of convalescent unvaccinated subjects as shown in **Figure 4B** (dashed lines). The difference in the IgA fraction is also evident when the results are grouped by virus variant (**Supplementary Figure 6**). Thus, IgA quantification reveals a behavior opposite with respect to total Ig levels.

### 3.5 Principal component analysis of Ig and IgA fingerprints

The combined quantification of Ig and IgA against SARS-COV-2 spike and nucleocapsid proteins as described above measured in the 27 subjects provides a set of 15 × 27 parameters that can be combined to further enhance the discrimination capability of the assay. To this aim, we performed a principal component analysis (PCA) of the whole data set of Ig and IgA data (**Supplementary Note S1**). The composition of the first three components (PC1, PC2, and PC3) is detailed in **Figure 4C**. As noticeable, PC1 approximately represents the difference between IgA and Ig levels, whereas PC2 roughly represents the average amount of Ig and IgA together. Surprisingly, PC3 is only related to *GU_IgA_* for delta and omicron variants.

In **Figure 4D**, we plot PC2 *vs.* PC1 for all samples, PC3 being represented by the size of the symbols. As apparent, the first two principal components are effective in separating vaccinated (blue symbols) from unvaccinated (red symbols) subjects. Interestingly, the spreading of the data along the third component spontaneously provides additional discrimination criteria for sample VX07, whose PC1 and PC2 values are instead similar to unvaccinated subjects.

We complemented PCA by performing data correlations, finding a positive average correlation among Ig and among IgA and a negative cross-correlation between the two groups (large total Ig are often associated with low levels of IgA), with the exception of anti-nucleocapsid Ig (**Supplementary Figures 7, 8**).

The effectiveness of PCA in spontaneously discriminating groups of individuals suggests that, in the presence of a larger set of data, our fingerprinting assay could be further strengthened by supervised analysis.

### 3.6 Evolution of immunoglobulin fingerprint of vaccinated subjects upon infection

**Figure 5:**
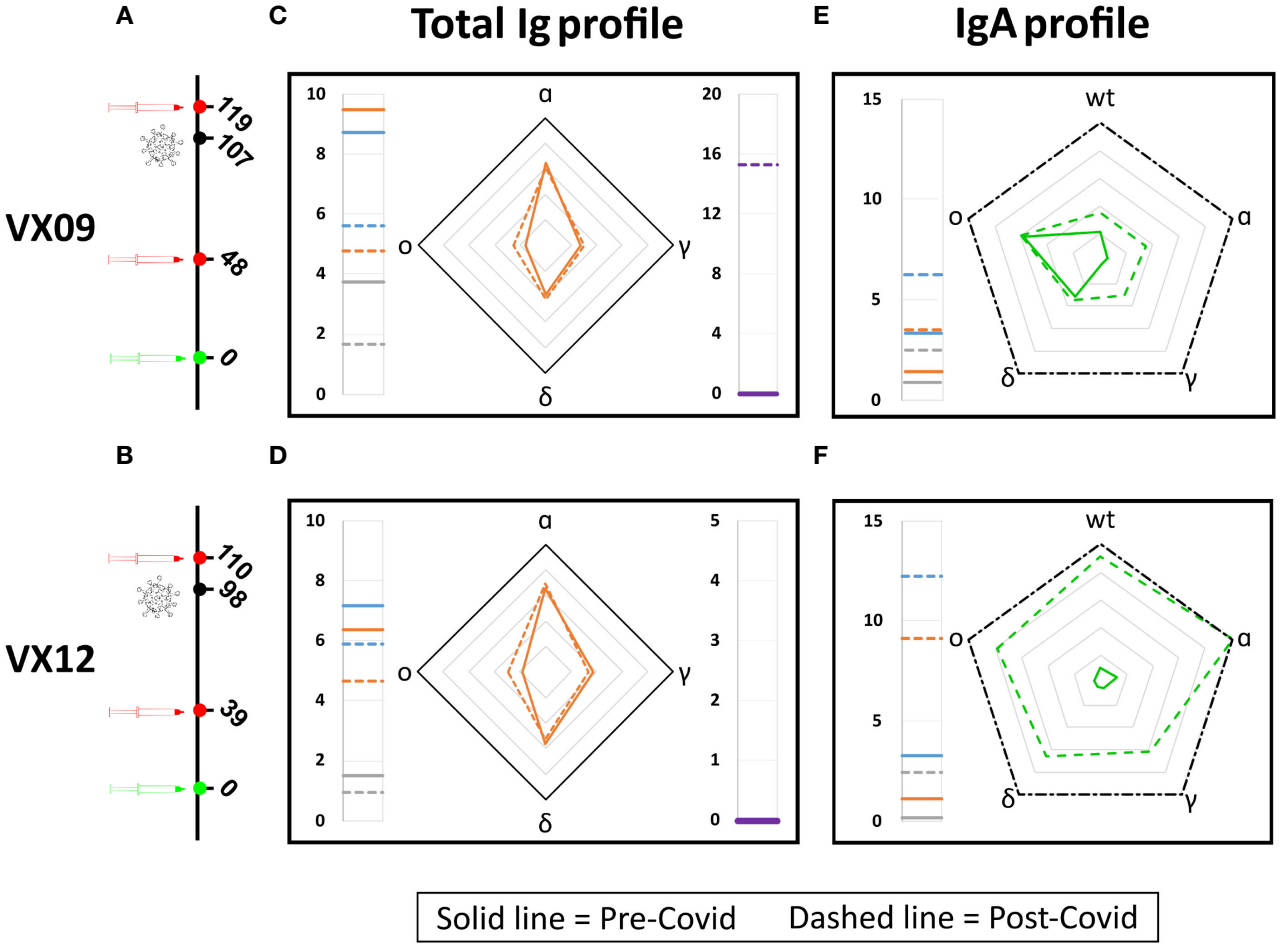
Effect of SARS-CoV-2 infection on antibody fingerprints of vaccinated subjects. Days from vaccination (green) of sample collections (red) and infection (black) for subjects VX09 **(A)** and VX12 **(B)**. Ig fingerprint (*RGU*) of subject VX09 **(C)** and VX12 **(D)** before (continuous lines) and after (dashed lines) a symptomatic infection with omicron variant. The legend for Ig fingerprints is reported in **Figure 2**. IgA fraction fingerprint (*GU_IgA_*) of subject VX09 **(E)** and VX12 **(F)** before (continuous lines) and after (dashed lines) the infection. The left side reports the reference quantification of IgA fraction in terms of *GU_IgA_* in units of 10^-4 s^-1 of three WT antigens: WT-spike protein (gray), WT-RBD (orange), and WT-LtRBD (blue). The radar chart reports the values of *GU_IgA_* for the WT, alpha, gamma, delta, and omicron RBD variants. The black dash-dotted contour line indicates *GU_IgA_* = 10^−3^ s^−1^.

To test the potential of the antigen array for detecting changes in the antibody fingerprints of a subject over time, we analyzed the *RGU* and *GU_IgA_* profile of two vaccinated subjects before and after a symptomatic COVID-19 infection due to the omicron variant (**Figure 5**). As shown in **Figures 5C, D**, for both subjects, the *RGU* profiles (radar chart) are very well maintained over time despite the different amounts of total Ig (side meters). Remarkably, the infection by the omicron variant only provided a small but clearly detectable increase in the amount of Ig binding to the omicron RBD. In contrast, the IgA fraction profiles (**Figures 5E, F**) did not show a relative increase for the omicron variant. The effect on IgA was a large increase in the overall amount for all variants, suggesting a significant but poorly specific IgA amplification upon infection.

## 4 Discussion

The Ig fingerprints obtained with our label-free antigen protein microarray demonstrate that the serum antibody repertoire can be analyzed up to single variant resolution, corresponding to RBD protein sequences differing by only a few amino acids (**Supplementary Figure 9**). Crucial to this result is the multiple internal references offered by multiplex label-free quantification (i.e., signal background, *σ*_0_, Δ*σ*(*t*_1_), and GU of WT variant).

The RPI assay here described is primarily based on the kinetics of binding, a choice that yields an important advantage since the parameter *GU_Ig_* can be quantified with a shorter measuring time than the affinity or the absolute concentration. This feature is relevant in the context of the development of rapid tests suitable for POC diagnostics. We found that the *GU_Ig_* profile strongly correlates with the variant of infection or vaccination. Differently from the commonly used absolute quantification of the IgG class of antibodies by ELISA, *GU_Ig_* measures the binding kinetics of total immunoglobulins. The correlation between the two quantities (shown in **Supplementary Figure 3**) is clear but not strong. Despite this, the relative *GU_Ig_* (i.e., *RGU*) profiles are found to be very stable with time, hence enabling the detection of small changes due to infections (**Figure 5**). The estimated limit of detection (LOD) of the proposed label-free microarray, obtained by comparing the quantification of total immunoglobulins against full spike protein and WT-RBD with that of anti-WT-RBD IgG measured by chemiluminescence enzyme immunoassay (**Supplementary Figure 10**), corresponds to 0.24 AU/mL for the antibodies against the full spike protein and 0.46 AU/mL for those against the RBD fragment, hence much lower than the typical value of 15 AU/mL considered as the positive response for the chemiluminescence assay (31).

Other novel technologies have been proposed to achieve the challenging task of combining rapidity and small LOD (32–34). The RPI biosensor used in this work brings the advantages of large multiplexing and cost-effective cartridge and set-up, suitable for large-scale production and POC testing. Nevertheless, the analysis here proposed can be effectively applied to any label-free biosensor with multiplex and kinetic measurement capabilities.

Characteristic response patterns for both Ig and IgA emerge for vaccinated and unvaccinated convalescent subjects, as confirmed by PCA. On average, vaccination induces higher levels of total Ig specific to the antigen, but virus infection produces higher levels of IgA, although less antigen-specific. This behavior is confirmed also considering the time-dependence of antibody levels after virus exposure (**Supplementary Figures 11, 12**). Another difference between the antibody repertoires of vaccinated and unvaccinated convalescent subjects is shown in **Figure 3D**, which suggests that SARS-CoV-2 infection yields a larger fraction of antibodies targeting full spike protein in regions different than RBD relative to vaccination. This is in agreement with previous work reporting a lower fraction of neutralizing antibodies in infected *vs.* vaccinated individuals, as well as a general correlation between IgG levels and neutralizing titers for the two groups of subjects (35). Indeed, as reported in **Supplementary Note 2**, the label-free microarray can be turned into a pseudo-neutralization assay by measuring the inhibition of the binding of the human angiotensin-converting enzyme 2 (ACE2) to the immobilized full spike and RBD proteins after incubation with a serum sample. Such pseudo-neutralization assay performed on a test sample (**Supplementary Figure 14**) shows a correlation between total Ig level profile and ACE2 binding inhibition, consistently with previous work.

Previous studies showed that early humoral response can be dominated by IgA antibodies, which can provide an important contribution to virus neutralization (36–40). Regarding the effect of vaccines on the levels of IgA, convalescent subjects were found to have larger levels of IgA than vaccinated subjects at a similar time after infection or vaccination (41–44). Our results are consistent with these observations and further support the difference in IgA levels between convalescent and vaccinated subjects so that the quantification of IgA levels could be exploited for viral infection screening and virus surveillance.

In conclusion, the proposed label-free antigen microarray demonstrates the feasibility of rapid serum antibody fingerprinting discriminating among single SARS-CoV-2 variants through the profile of total Ig levels. The results obtained suggest that this method may be useful for the serological recognition of infecting viral variants even in subjects with low viral loads or who have already eliminated the virus. A limitation of the study is given by the small number of subjects infected by specific virus variants. However, the same approach can be applied on a larger scale to assess the epidemiology of SARS-CoV-2 infection and can be instrumental in planning strategies for control measures in the future. Another limitation of the study is that, in addition to total Ig, only the IgA class of immunoglobulins was investigated, finding no clear correlation with the variant of infection in this case. Therefore, which antibody class or subclass primarily contributes to the observed dependence of total Ig fingerprint on virus variant remains undetermined. Our results may represent the basis for further investigations on the application of this method in contexts where it may be important to retrospectively reconstruct the infecting viral genotype in already recovered individuals or patients with insufficient viral nucleic acid amounts for genotyping (45), such as in the case of HCV or HIV or flaviviruses (46–48).

## Data availability statement

The original contributions presented in the study are included in the article/**Supplementary Material.** Further inquiries can be directed to the corresponding authors.

## Ethics statement

The studies involving humans were approved by ASST Fatebenefratelli Sacco, Milano, Italy, 293, protocol No. 379/2020 of March 2020, then amended in January 2023. The studies were conducted in accordance with the local legislation and institutional requirements. The participants provided their written informed consent to participate in this study.

## Author contributions

TC: Data curation, Formal analysis, Investigation, Methodology, Visualization, Writing – review & editing. LC: Data curation, Investigation, Methodology, Visualization, Writing – review & editing. GN: Data curation, Formal analysis, Visualization, Writing – review & editing. GZa: Conceptualization, Investigation, Writing – review & editing. TI: Data curation, Formal analysis, Writing – review & editing. MC: Methodology, Resources, Writing – review & editing. VB: Conceptualization, Investigation, Writing – review & editing. SE: Conceptualization, Resources, Writing – review & editing. CB: Conceptualization, Funding acquisition, Resources, Writing – review & editing. AL: Investigation, Methodology, Resources, Validation, Writing – review & editing. GZe: Investigation, Methodology, Resources, Validation, Writing – review & editing. TB: Conceptualization, Funding acquisition, Writing – original draft, Writing – review & editing, Resources, Supervision. MB: Conceptualization, Funding acquisition, Resources, Supervision, Writing – original draft, Writing – review & editing, Investigation.

## References

[1] Wine Y Horton AP Ippolito GC Georgiou G. Serology in the 21st century: the molecular-level analysis of the serum antibody repertoire. Curr Opin Immunol. (2015) 35:89–97. doi: 10.1016/j.coi.2015.06.009 26172290 PMC4553097

[2] Ionov S Lee J. An immunoproteomic survey of the antibody landscape: Insights and opportunities revealed by serological repertoire profiling. Front Immunol. (2022) 13:832533. doi: 10.3389/fimmu.2022.832533 35178051 PMC8843944

[3] Byrnes JR Zhou XX Lui I Elledge SK Glasgow JE Lim SA. Competitive SARS-CoV-2 serology reveals most antibodies targeting the spike receptor-binding domain compete for ACE2 binding. mSphere. (2020) 5:e00802. doi: 10.1128/msphere.00802-20 PMC7494835 32938700

[4] Johnson M Wagstaffe HR Gilmour KC Mai AL Lewis J Hunt A. Evaluation of a novel multiplexed assay for determining IgG levels and functional activity to SARS-CoV-2. J Clin Virol. (2020) 130:104572. doi: 10.1016/j.jcv.2020.104572 32769024 PMC7396134

[5] Castillo-Olivares J Wells DA Ferrari M Chan ACY Smith P Nadesalingam A. Analysis of serological biomarkers of SARS-CoV-2 infection in convalescent samples from severe, moderate and mild COVID-19 cases. Front Immunol. (2021) 12:748291. doi: 10.3389/fimmu.2021.748291 34867975 PMC8640495

[6] Garcia-Beltran WF Lam EC Denis KS Nitido AD Garcia ZH Hauser BM. Multiple SARS-CoV-2 variants escape neutralization by vaccine-induced humoral immunity. Cell. (2021) 184:2372–2383.e9. doi: 10.1016/j.cell.2021.03.013 33743213 PMC7953441

[7] Harvey WT Carabelli AM Jackson B Gupta RK Thomson EC Harrison EM. SARS-CoV-2 variants, spike mutations and immune escape. Nat Rev Microbiol. (2021) 19:409–24. doi: 10.1038/s41579-021-00573-0 PMC8167834 34075212

[8] Ong DS Fragkou PC Schweitzer VA Chemaly RF Moschopoulos CD Skevaki C. How to interpret and use COVID-19 serology and immunology tests. Clin Microbiol Infection. (2021) 27:981–6. doi: 10.1016/j.cmi.2021.05.001 PMC8106522 33975005

[9] Siracusano G Brombin C Pastori C Cugnata F Noviello M Tassi E. Profiling antibody response patterns in COVID-19: Spike S1-reactive IgA signature in the evolution of SARS-CoV-2 infection. Front Immunol. (2021) 12:772239. doi: 10.3389/fimmu.2021.772239 34804064 PMC8595940

[10] Wei J Matthews PC Stoesser N Maddox T Lorenzi L Studley R. Anti-spike antibody response to natural SARS-CoV-2 infection in the general population. Nat Commun. (2021) 12:6250. doi: 10.1038/s41467-021-26479-2 34716320 PMC8556331

[11] Criscuolo E Diotti RA Strollo M Rolla S Ambrosi A Locatelli M. Weak correlation between antibody titers and neutralizing activity in sera from SARS-CoV-2 infected subjects. J Med Virol. (2020) 93:2160–7. doi: 10.1002/jmv.26605 PMC7675753 33064340

[12] Peeling RW Wedderburn CJ Garcia PJ Boeras D Fongwen N Nkengasong J. Serology testing in the COVID-19 pandemic response. Lancet Infect Dis. (2020) 20:e245–9. doi: 10.1016/S1473-3099(20)30517-X PMC7367660 32687805

[13] Dörschug A Schwanbeck J Hahn A Hillebrecht A Blaschke S Mese K. Comparison of five serological assays for the detection of SARS-CoV-2 antibodies. Diagnostics. (2021) 11:78. doi: 10.3390/diagnostics11010078 33418886 PMC7825051

[14] Ripperger TJ Uhrlaub JL Watanabe M Wong R Castaneda Y Pizzato HA. Orthogonal SARS-CoV-2 serological assays enable surveillance of low-prevalence communities and reveal durable humoral immunity. Immunity. (2020) 53:925–933.e4. doi: 10.1016/j.immuni.2020.10.004 33129373 PMC7554472

[15] Weber LK Palermo A Kügler J Armant O Isse A Rentschler S. Single amino acid fingerprinting of the human antibody repertoire with high density peptide arrays. J Immunol Methods. (2017) 443:45–54. doi: 10.1016/j.jim.2017.01.012 28167275

[16] Paull ML Daugherty PS. Mapping serum antibody repertoires using peptide libraries. Curr Opin Chem Eng. (2018) 19:21–6. doi: 10.1016/j.coche.2017.12.001

[17] Cheng L Zhang X Chen Y Wang D Zhang D Yan S. Dynamic landscape mapping of humoral immunity to SARS-CoV-2 identifies non-structural protein antibodies associated with the survival of critical COVID-19 patients. Signal Transduction Targeted Ther. (2021) 6:304. doi: 10.1038/s41392-021-00718-w PMC8368053 34404759

[18] Heffron AS McIlwain SJ Amjadi MF Baker DA Khullar S Armbrust T. The landscape of antibody binding in SARS-CoV-2 infection. PloS Biol. (2021) 19:e3001265. doi: 10.1371/journal.pbio.3001265 34143766 PMC8245122

[19] Mishra N Huang X Joshi S Guo C Ng J Thakkar R. Immunoreactive peptide maps of SARS-CoV-2. Commun Biol. (2021) 4:225. doi: 10.1038/s42003-021-01743-9 33580175 PMC7881038

[20] Jiang H Li Y Zhang H Wang W Yang X Qi H. SARS-CoV-2 proteome microarray for global profiling of COVID-19 specific IgG and IgM responses. Nat Commun. (2020) 11:3581. doi: 10.1038/s41467-020-17488-8 32665645 PMC7360742

[21] Berre ML Paulovčáková T Verissimo CDM Doyle S Dalton JP Masterson C. A new multiplex SARS-CoV-2 antigen microarray showed correlation of IgG, IgA, and IgM antibodies from patients with COVID-19 disease severity and maintenance of relative IgA and IgM antigen binding over time. PloS One. (2023) 18:e0283537. doi: 10.1371/journal.pone.0283537 36996259 PMC10062637

[22] Fink S Ruoff F Stahl A Becker M Kaiser P Traenkle B. Multiplexed serum antibody screening platform using virus extracts from endemic coronaviridae and SARS-CoV-2. ACS Infect Dis. (2021) 7:1596–606. doi: 10.1021/acsinfecdis.0c00725 33724771

[23] Giavazzi F Salina M Cerbino R Bassi M Prosperi D Ceccarello E. Multispot, label-free biodetection at a phantom plastic–water interface. Proc Natl Acad Sci. (2013) 110:9350–5. doi: 10.1073/pnas.1214589110 PMC3677498 23696673

[24] Salina M Giavazzi F Lanfranco R Ceccarello E Sola L Chiari M. Multispot, label-free immunoassay on reflectionless glass. Biosensors Bioelectronics. (2015) 74:539–45. doi: 10.1016/j.bios.2015.06.064 26188676

[25] Varotto-Boccazzi I Manenti A Dapporto F Gourlay LJ Bisaglia B Gabrieli P. Epidemic preparedness-leishmania tarentolae as an easy-to-handle tool to produce antigens for viral diagnosis: Application to COVID-19. Front Microbiol. (2021) 12:736530. doi: 10.3389/fmicb.2021.736530. 34966362 PMC8710741

[26] Wrapp D Wang N Corbett KS Goldsmith JA Hsieh C-L Abiona O. Cryo-EM structure of the 2019-nCoV spike in the prefusion conformation. Science. (2020) 367:1260–3. doi: 10.1126/science.abb2507 PMC7164637 32075877

[27] Schmitz A Weber A Bayin M Breuers S Fieberg V Famulok M. A SARS-CoV-2 spike binding DNA aptamer that inhibits pseudovirus infection by an rbd-independent mechanism. Angewandte Chemie Int Edition. (2021) 60:10279–85. doi: 10.1002/anie.202100316 PMC8251191 33683787

[28] Vanjur L Carzaniga T Casiraghi L Zanchetta G Damin F Sola L. Copolymer coatings for DNA biosensors: Effect of charges and immobilization chemistries on yield, strength and kinetics of hybridization. Polymers. (2021) 13(22):3897. doi: 10.3390/polym13223897 34833198 PMC8625010

[29] Cretich M Pirri G Damin F Solinas I Chiari M. A new polymeric coating for protein microarrays. Anal Biochem. (2004) 332:67–74. doi: 10.1016/j.ab.2004.05.041. 15301950

[30] Wheeler SE Shurin GV Yost M Anderson A Pinto L Wells A. Differential antibody response to mRNA COVID-19 vaccines in healthy subjects. Microbiol Spectr. (2021) 9:e0034121. doi: 10.1128/Spectrum.00341-21. 34346750 PMC8552678

[31] Nguyen NN Mutnal MB Gomez RR Pham HN Nguyen LT Koss W. Correlation of ELISA method with three other automated serological tests for the detection of anti-SARS-CoV-2 antibodies. PloS One. (2020) 15:e0240076. doi: 10.1371/journal.pone.0240076 33022019 PMC7537879

[32] Cardoso AR Cabral-Miranda G Reyes-Sandoval A Bachmann MF Sales MGF. Detecting circulating antibodies by controlled surface modification with specific target proteins: Application to malaria. Biosensors Bioelectronics. (2017) 91:833–41. doi: 10.1016/j.bios.2017.01.031 28157657

[33] Calvo-Lozano O Sierra M Soler M Estévez MC Chiscano-Camón L Ruiz-Sanmartin A. Label-free plasmonic biosensor for rapid, quantitative, and highly sensitive COVID-19 serology: Implementation and clinical validation. Analytical Chem. (2021) 94:975–84. doi: 10.1021/acs.analchem.1c03850 PMC8751014 34971311

[34] Zhao B Che C Wang W Li N Cunningham BT. Single-step, wash-free digital immunoassay for rapid quantitative analysis of serological antibody against SARS-CoV-2 by photonic resonator absorption microscopy. Talanta. (2021) 225:122004. doi: 10.1016/j.talanta.2020.122004 33592744 PMC7833826

[35] Manenti A Gianchecchi E Dapporto F Leonardi M Cantaloni P Fattorini F. Evaluation and correlation between SARS-CoV-2 neutralizing and binding antibodies in convalescent and vaccinated subjects. J Immunol Methods. (2022) 500:113197. doi: 10.1016/j.jim.2021.113197 34843712 PMC8619878

[36] Chen K Magri G Grasset EK Cerutti A. Rethinking mucosal antibody responses: IgM, IgG and IgD join IgA. Nat Rev Immunol. (2020) 20:427–41. doi: 10.1038/s41577-019-0261-1 PMC10262260 32015473

[37] Padoan A Sciacovelli L Basso D Negrini D Zuin S Cosma C. IgA-ab response to spike glycoprotein of SARS-CoV-2 in patients with COVID-19: A longitudinal study. Clinica Chimica Acta. (2020) 507:164–6. doi: 10.1016/j.cca.2020.04.026 PMC7194886 32343948

[38] Sterlin D Mathian A Miyara M Mohr A Anna F Claër L. IgA dominates the early neutralizing antibody response to SARS-CoV-2. Sci Trans Med. (2021) 13(557):eabd2223. doi: 10.1126/scitranslmed.abd2223 PMC7857408 33288662

[39] Zervou FN Louie P Stachel A Zacharioudakis IM Ortiz-Mendez Y Thomas K. SARS-CoV-2 antibodies: IgA correlates with severity of disease in early COVID-19 infection. J Med Virol. (2021) 93:5409–15. doi: 10.1002/jmv.27058 PMC8242647 33932299

[40] Havervall S Marking U Svensson J Greilert-Norin N Bacchus P Nilsson P. Anti-spike mucosal IgA protection against SARS-CoV-2 omicron infection. New Engl J Med. (2022) 387:1333–6. doi: 10.1056/nejmc2209651 PMC9511632 36103621

[41] Wisnewski AV Luna JC Redlich CA. Human IgG and IgA responses to COVID-19 mRNA vaccines. PloS One. (2021) 16:e0249499. doi: 10.1371/journal.pone.0249499 34133415 PMC8208542

[42] Cheng ZJ Zheng P Xue M Chen Y Sun B. Identifying COVID-19 infections from a vaccinated population using specific IgA antibody test. Front Immunol. (2022) 13:821218. doi: 10.3389/fimmu.2022.821218 35173731 PMC8841746

[43] Sano K Bhavsar D Singh G Floda D Srivastava K Gleason C. SARS-CoV-2 vaccination induces mucosal antibody responses in previously infected individuals. Nat Commun. (2022) 13:5135. doi: 10.1038/s41467-022-32389-8 36050304 PMC9435409

[44] Sheikh-Mohamed S Isho B Chao GY Zuo M Cohen C Lustig Y. Systemic and mucosal IgA responses are variably induced in response to SARS-CoV-2 mRNA vaccination and are associated with protection against subsequent infection. Mucosal Immunol. (2022) 15:799–808. doi: 10.1038/s41385-022-00511-0 35468942 PMC9037584

[45] Mannarino S Lanna M Calcaterra V Carzaniga T Casiraghi L Lai A. Fetal myocarditis associated with maternal SARS-CoV-2 infection. Pediatr Infect Dis J. (2024). doi: 10.1097/inf.0000000000004245 38190639

[46] Pawlotsky JM Prescott L Simmonds P Pellet C Laurent-Puig P Labonne C. Serological determination of hepatitis c virus genotype: comparison with a standardized genotyping assay. J Clin Microbiol. (1997) 35:1734–9. doi: 10.1128/jcm.35.7.1734-1739.1997 PMC229831 9196183

[47] Murphy G Belda FJ Pau C-P Clewley JP Parry JV. Discrimination of subtype b and non-subtype b strains of human immunodeficiency virus type 1 by serotyping: Correlation with genotyping. J Clin Microbiol. (1999) 37:1356–60. doi: 10.1128/jcm.37.5.1356-1360.1999 PMC84775 10203486

[48] Cleton NB Godeke G-J Reimerink J Beersma MF van Doorn HR Franco L. Spot the difference—development of a syndrome based protein microarray for specific serological detection of multiple flavivirus infections in travelers. PloS Negl Trop Dis. (2015) 9:e0003580. doi: 10.1371/journal.pntd.0003580 25767876 PMC4359159

