# Supplementary material for: Serum antibody fingerprinting of SARS-CoV-2 variants in infected and vaccinated subjects by label-free microarray biosensor
Source: Front Immunol. 2024 Feb 27;15:1323406. doi: 10.3389/fimmu.2024.1323406 (PMC10927789; doi:10.3389/fimmu.2024.1323406)
Supplement: Supplementary file 1 [file DataSheet_1.pdf]

Serum antibody fingerprinting of SARS-CoV-2 variants in infected and vaccinated subjects by label-free microarray biosensor

Supplementary Material

1 SUPPLEMENTARY TABLES AND FIGURES

1.1 Supplementary Table 1

Table S1. Virus strain and sample collection time for samples of convalescent subjects.

Code	SARS-CoV-2 strain	Variant	Onset of symptoms	Plasma collection date	Days from symptoms
NV01	B.1.177	WT-ES	11/2020	11/2020	2
NV02	B.1	WT	02/2020	02/2020	3
NV03	BA.1	omicron 1	01/2022	01/2022	4
NV04	B.1.177	WT-ES	02/2021	02/2021	4
NV05	B.1	WT	02/2020	02/2020	8
NV06	B.1	WT	02/2020	02/2020	11
NV07	B.1.1.7	alpha	05/2021	05/2021	11
NV08	B.1.1.7	alpha	05/2021	05/2021	12
NV09	P.1	gamma	06/2021	06/2021	12
NV10	BA.2	omicron 2	03/2022	03/2022	15
NV11	B.1.617.2	delta	09/2021	10/2021	17
NV12	B.1.177	WT-ES	03/2021	04/2021	20
NV13	B.1.1.7	alpha	05/2021	06/2021	22
VX09b	BA.2	omicron 2	04/2022	04/2022	12
VX12b	BA.2	omicron 2	04/2022	04/2022	12

1.2 Supplementary Table 2

Table S2. Virus strain and sample collection time for samples of vaccinated subjects.

Code	Vaccine 1st dose	Vaccine 2nd dose	Date 2nd dose	Plasma Collection date	Days from 2nd dose
VX01	BNT162b2 (Comirnaty)	BNT162b2 (Comirnaty)	02/2021	02/2021	9
VX02	BNT162b2 (Comirnaty)	BNT162b2 (Comirnaty)	02/2021	02/2021	11
VX03	Vaxzevria (ChAdOx1-S)	Vaxzevria (ChAdOx1-S)	01/2022	02/2021	15
VX04	BNT162b2 (Comirnaty)	BNT162b2 (Comirnaty)	02/2021	02/2021	16
VX05	BNT162b2 (Comirnaty)	BNT162b2 (Comirnaty)	02/2021	02/2021	16
VX06	BNT162b2 (Comirnaty)	BNT162b2 (Comirnaty)	01/2021	02/2021	20
VX07	BNT162b2 (Comirnaty)	BNT162b2 (Comirnaty)	01/2021	02/2021	22
VX08	BNT162b2 (Comirnaty)	BNT162b2 (Comirnaty)	01/2022	02/2022	29
VX09	Vaxzevria (ChAdOx1-S)	BNT162b2 (Comirnaty)	12/2021	02/2022	39
VX10	Vaxzevria (ChAdOx1-S)	Vaxzevria (ChAdOx1-S)	12/2021	02/2022	41
VX11	Vaxzevria (ChAdOx1-S)	BNT162b2 (Comirnaty)	12/2021	02/2022	44
VX12	Vaxzevria (ChAdOx1-S)	Vaxzevria (ChAdOx1-S)	12/2021	02/2022	48
VX13	Vaxzevria (ChAdOx1-S)	Vaxzevria (ChAdOx1-S)	11/2021	02/2022	63
VX14	BNT162b2 (Comirnaty)	BNT162b2 (Comirnaty)	10/2021	02/2022	103

.

1.3 Supplementary Figure 1

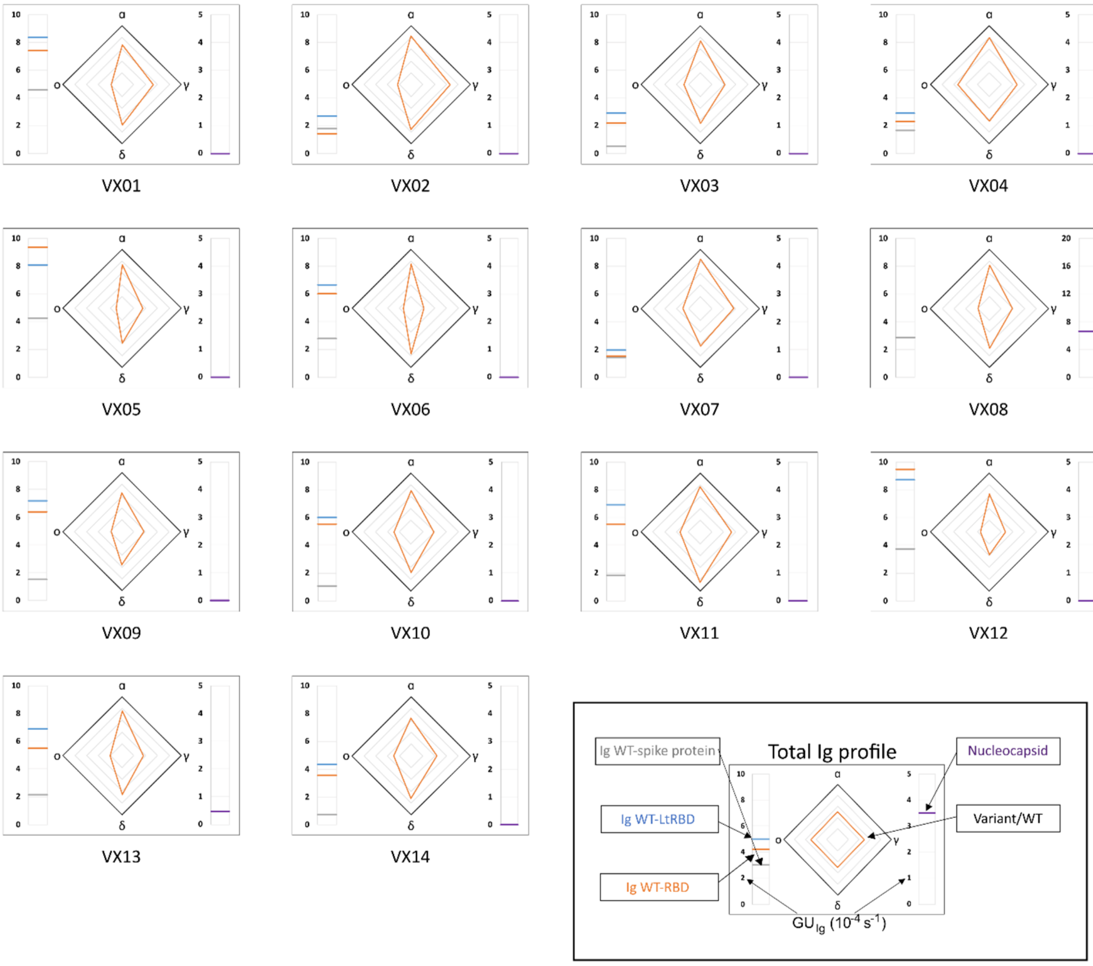

Figure S1. Immunoglobulins fingerprints of all samples of vaccinated subjects. The legend of the fingerprint diagram is shown in the box at the bottom of the figure. The left side reports the quantification of Ig in terms of GU$_{Ig}$ of three WT antigens, as indicated. The right side reports the quantification of anti-nucleocapsid antibodies expressed as GU$_{Ig}$. The radar chart reports the values of RGU for the alpha, gamma, delta and omicron RBD variants. The thick black contour line represents the reference amount of antibodies binding to WT-RBD hence corresponding to RGU = 1.

1.4 Supplementary Figure 2

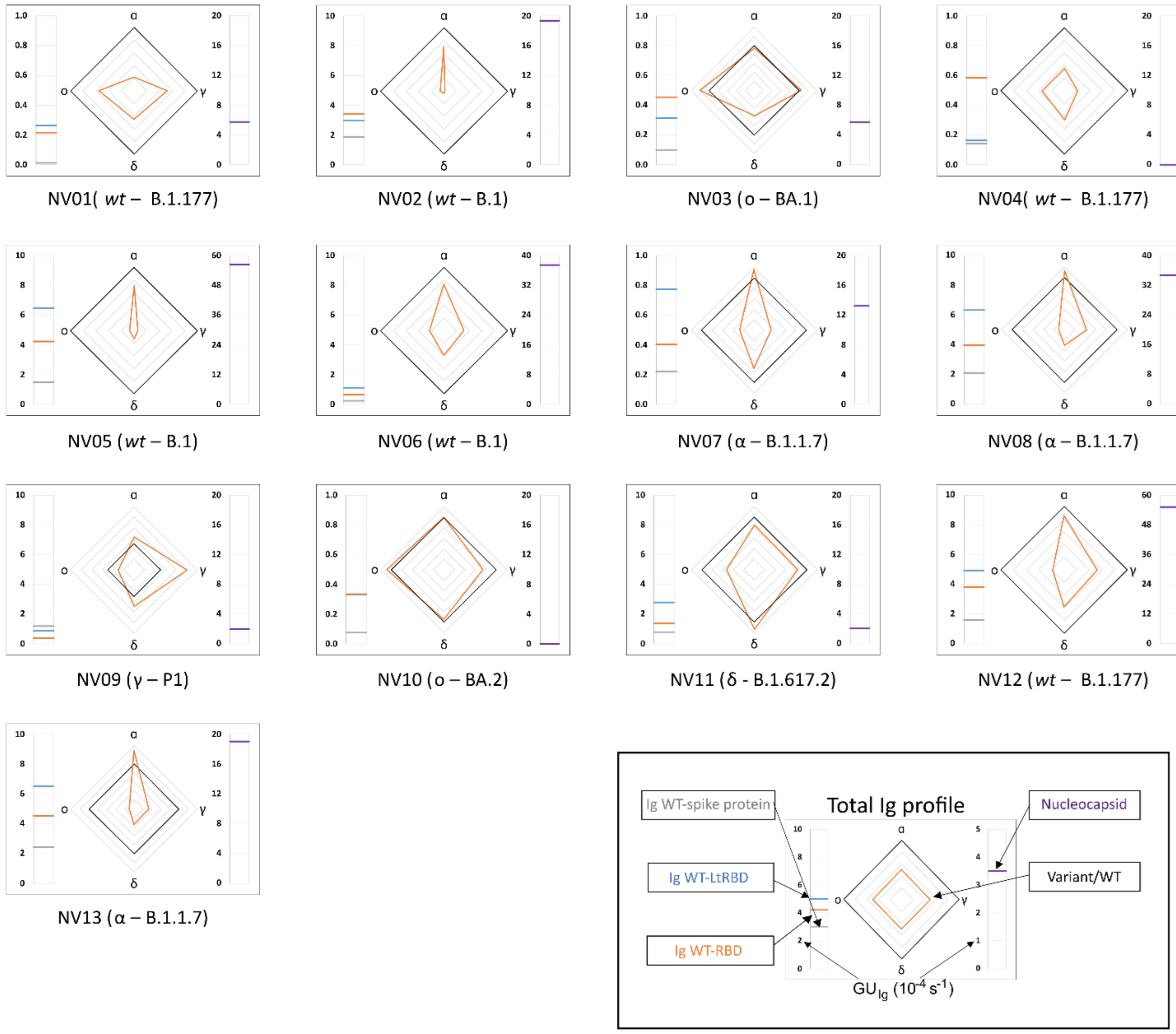

Figure S2. Immunoglobulins fingerprints of all samples of convalescent unvaccinated subjects. The legend of the fingerprint diagram is shown in the box at the bottom of the figure. The left side reports the quantification of Ig in terms of GU_{Ig} of three WT antigens, as indicated. The right side reports the quantification of anti-nucleocapsid antibodies expressed as GU_{Ig}. The radar chart reports the values of RGU for the alpha, gamma, delta and omicron RBD variants. The thick black contour line represents the reference amount of antibodies binding to WT-RBD hence corresponding to RGU = 1.

1.5 Supplementary Figure 3

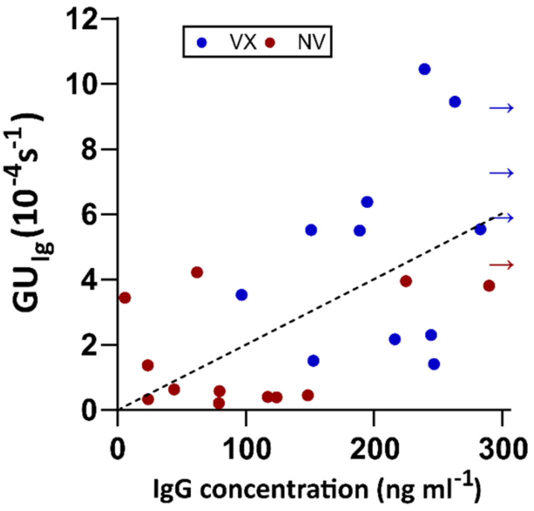

Figure S3. Quantification of IgG by ELISA. The growth units of total immunoglobulins obtained by label-free microarray are plotted as a function of the concentration of IgG estimated by ELISA for vaccinated (blue) and unvaccinated (red) subjects. The arrows indicate data points of GU_{Ig} exceeding the saturation level for ELISA quantification. The dashed line corresponds to a linear fit with slope 0.02 s^{-1}/(ng mL^{-1}).

1.6 Supplementary Figure 4

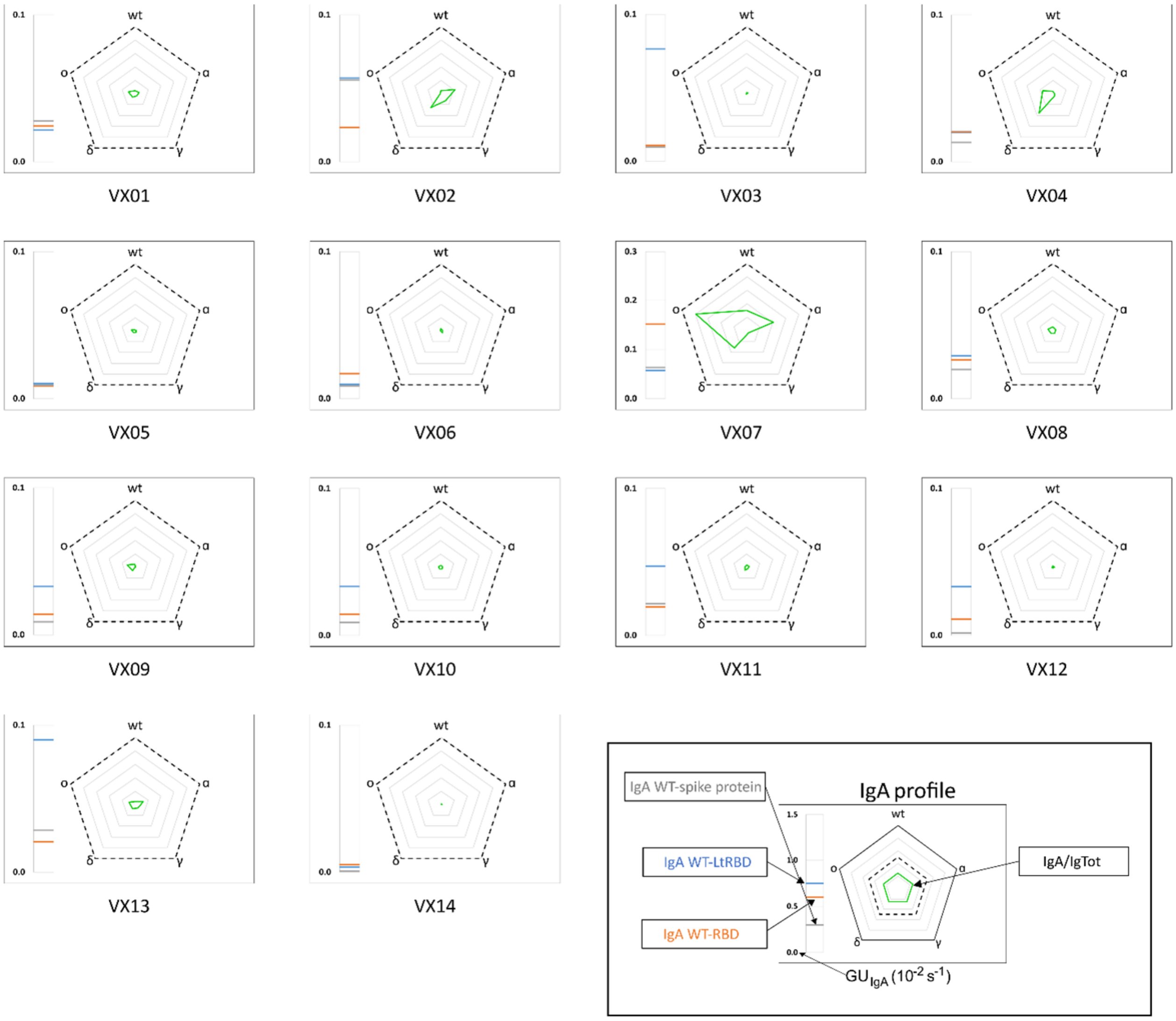

Figure S4. Fingerprint of IgA fraction against different SARS-CoV-2 antigen variants of all samples of vaccinated subjects. The legend of the fingerprint diagram is shown in the box at the bottom of the figure. The left side reports the reference quantification of IgA fraction in terms of GU_{IgA} of three WT antigens, as indicated. The radar chart reports the values of GU_{IgA} for the WT, alpha, gamma, delta and omicron RBD variants. The thick black contour line and the dashed contour line indicate $GU_{IgA} = 10^{-2}$ s^{-1} and $GU_{IgA} = 0.5 \cdot 10^{-2}$ s^{-1} , respectively.

1.7 Supplementary Figure 5

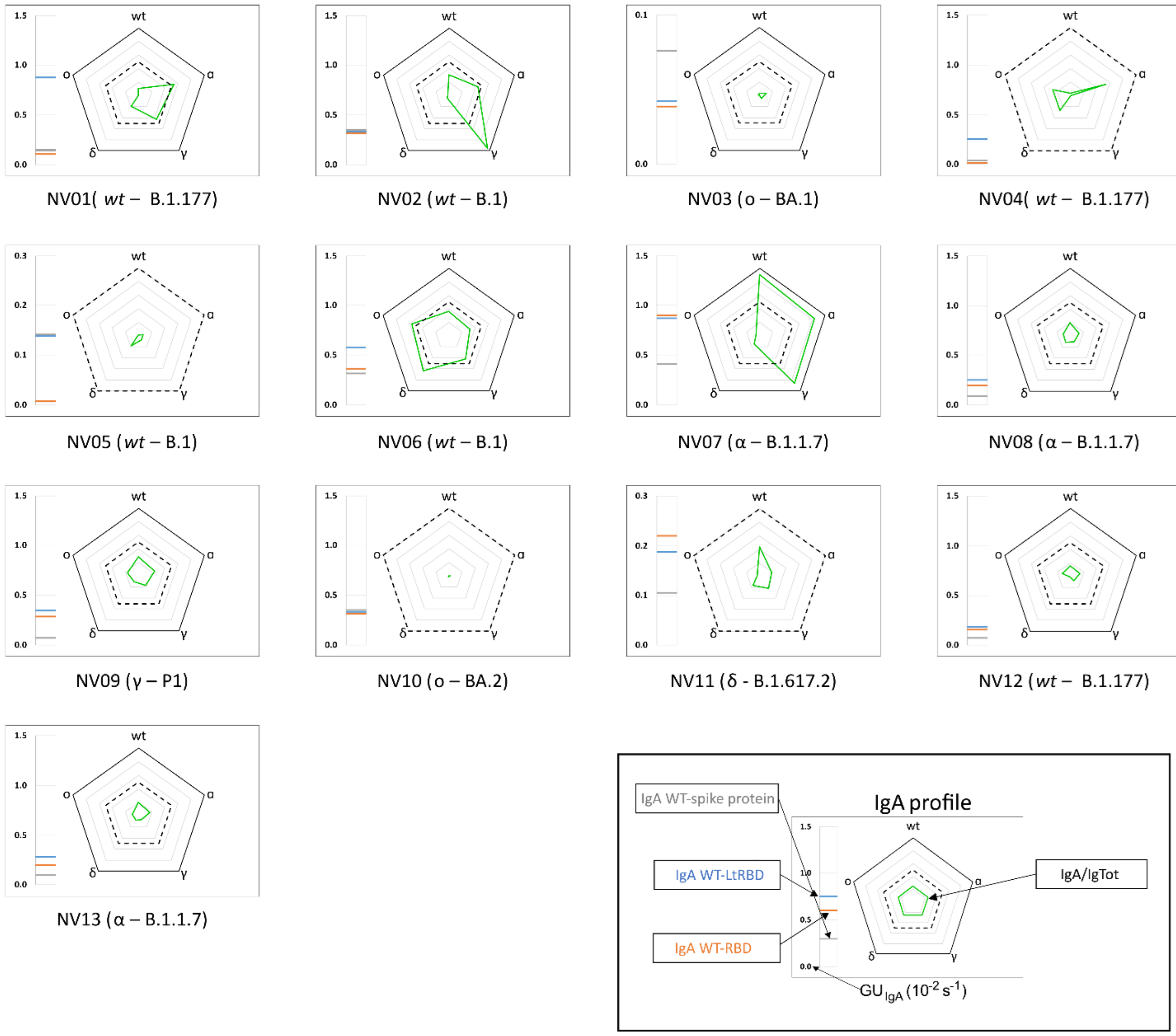

Figure S5. Fingerprint of IgA fraction against different SARS-CoV-2 antigen variants of all samples of convalescent unvaccinated subjects. The legend of the fingerprint diagram is shown in the box at the bottom of the figure. The left side reports the reference quantification of IgA fraction in terms of GU_{IgA} of three WT antigens, as indicated. The radar chart reports the values of GU_{IgA} for the WT, alpha, gamma, delta and omicron RBD variants. The thick black contour line and the dashed contour line indicate $GU_{IgA} = 10^{-2}\ s^{-1}$ and $GU_{IgA} = 0.5 \cdot 10^{-2}\ s^{-1}$, respectively.

1.8 Supplementary Figure 6

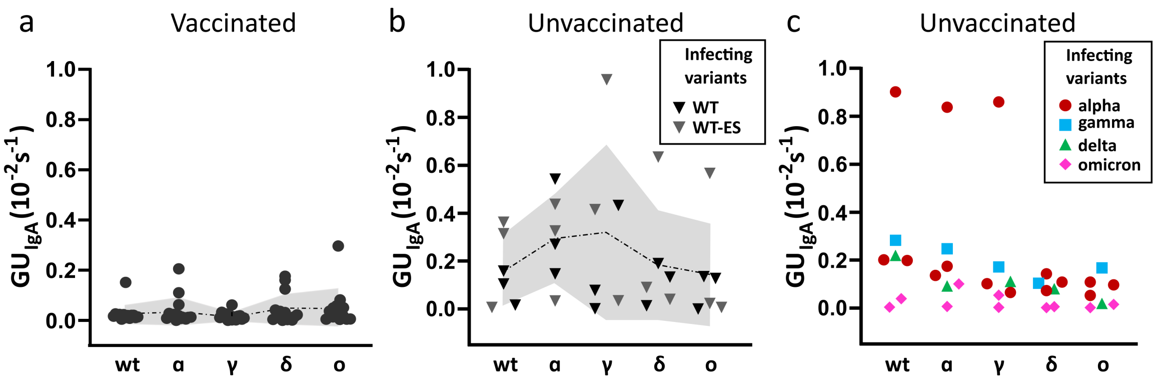

Figure S6. Fingerprint of IgA fraction against different SARS-CoV-2 antigen variants of vaccinated and unvaccinated convalescent subjects. (a) Summary of GU_{IgA} measured for plasma samples of vaccinated subjects. (b) Summary of GU_{IgA} measured for plasma samples of unvaccinated convalescent subjects. The color of the data points refers to samples infected with strain B.1 (WT) or B.1.177 (WT-ES) as indicated in the legend. (c) Summary of GU_{IgA} measured for plasma samples of unvaccinated convalescent subjects infected with the variants indicated in the legend. In panels a and b, the dashed line and the shaded area connect the average values and the standard deviation values of the data points, respectively.

1.9 Supplementary Figure 7

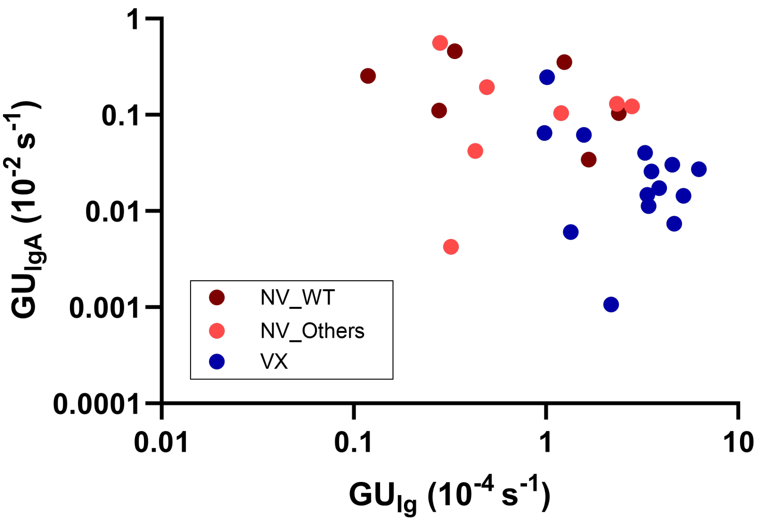

Figure S7. Scatter plot for IgA fraction versus Ig levels for all RBD variants. The average values of GU_{IgA} measured for all RBD variants are plotted as a function of the average values of GU_{Ig} measured for all RBD variants. The colour of the data point refers to vaccinated subjects (blue) and unvaccinated convalescent subjects infected with WT variant (dark red) or one of the other variants (light red).

1.10 Supplementary Figure 8

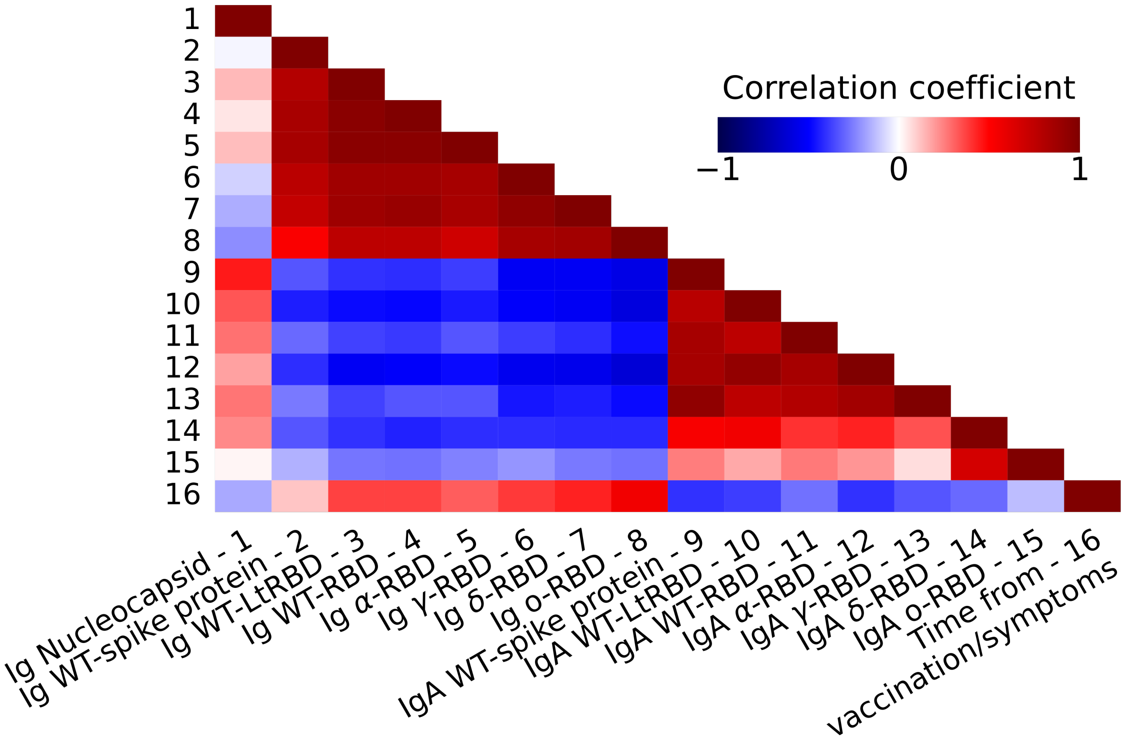

Figure S8. Correlation matrix between all Ig and IgA quantification parameters, expressed by GU_{Ig} and GU_{IgA}, respectively. The correlation matrix also includes the time (days) passed after vaccination or infection. The Figure shows that Ig and IgA quantification are anti-correlated for all variants, meaning that high levels of total Ig is often associated to low levels if IgA and vice versa. Additionally, the levels of total Ig are all correlated among them, whereas the correlations among the IgA levels are more heterogeneous, being GU_{IgA} for delta and omicron variants weakly correlated with the WT, alpha and gamma variants. Similarly, anti-nucleocapsid Ig levels are weakly or anti-correlated with anti-RBD and anti-spike protein Ig, but the correlation with IgA levels is larger for all variants except for omicron. The time from vaccination or symptoms shows a positive correlation with Ig levels and negative with IgA. This behaviour is ascribed to the rather short interval between infection or vaccination and sample collection of the analysed samples (Supplementary Tables 1 and 2).

1.11 Supplementary Figure 9

a

```
WT-RBD          RVQPTESIVRFPNITNLCPFGEVFNATRFASVYAWNRKRISNCVADYSVLYNSASFSTFK    60
alpha-RBD       RVQPTESIVRFPNITNLCPFGEVFNATRFASVYAWNRKRISNCVADYSVLYNSASFSTFK    60
gamma-RBD       RVQPTESIVRFPNITNLCPFGEVFNATRFASVYAWNRKRISNCVADYSVLYNSASFSTFK    60
delta-RBD       RVQPTESIVRFPNITNLCPFGEVFNATRFASVYAWNRTRISNCVADYSVLYNSASFSTFK    60
omicron-RBD     RVQPTESIVRFPNITNLCPFDEVFNATRFASVYAWNRKRISNCVADYSVLYNLAPFFTFK    60
                ********************.****************.************** * * ***

WT-RBD          CYGVSPTKLNDLCFTNVYADSFVIRGDEVRQIAPGQTGKIADYNYKLPDDFTGCVIAWNS    120
alpha-RBD       CYGVSPTKLNDLCFTNVYADSFVIRGDEVRQIAPGQTGKIADYNYKLPDDFTGCVIAWNS    120
gamma-RBD       CYGVSPTKLNDLCFTNVYADSFVIRGDEVRQIAPGQTGKIADYNYKLPDDFTGCVIAWNS    120
delta-RBD       CYGVSPTKLNDLCFTNVYADSFVIRGDEVRQIAPGQTGKIADYNYKLPDDFTGCVIAWNS    120
omicron-RBD     CYGVSPTKLNDLCFTNVYADSFVIRGDEVRQIAPGQTGNIADYNYKLPDDFTGCVIAWNS    120
                **************************************:*********************

WT-RBD          NNLDSKVGGNYNYLYRLFRKSNLKPFERDISTEIYQAGSTPCNGVEGFNCYFPLQSYGFQ    180
alpha-RBD       NNLDSKVGGNYNYLYRLFRKSNLKPFERDISTEIYQAGSTPCNGVEGFNCYFPLQSYGFQ    180
gamma-RBD       NNLDSKVGGNYNYRYRLFRKSNLKPFERDISTEIYQAGSKPCNGVEGFNCYFPLQSYGFQ    180
delta-RBD       NNLDSKVGGNYNYLYRLFRKSNLKPFERDISTEIYQAGSTPCNGVKGFNCYFPLQSYGFQ    180
omicron-RBD     NKLDSKVSGNYNYLYRLFRKSNLKPFERDISTEIYQAGNKPCNGVAGFNCYFPLRSYSFR    180
                *:*****.***** ************************..***** ********:**.*:

WT-RBD          PTNGVGYQPYRVVVLSFELLHAPATVCGPKKSTNLVKNKCVNF       223
alpha-RBD       PTYGVGYQPYRVVVLSFELLHAPATVCGPKKSTNLVKNKCVNF       223
gamma-RBD       PTNGVGYQPYRVVVLSFELLHAPATVCGPKKSTNLVKNKCVNF       223
delta-RBD       PTYGVGYQPYRVVVLSFELLHAPATVCGPKKSTNLVKNKCVNF       223
omicron-RBD     PTYGVGHQPYRVVVLSFELLHAPATVCGPKKSTNLVKNKCVNF       223
                ** ***:************************************
```

b

	WT-RBD	alpha-RBD	gamma-RBD	delta-RBD	omicron-RBD
**WT-RBD**	0	1	3	2	15
**alpha-RBD**	1	0	2	3	14
**gamma-RBD**	3	2	0	5	16
**delta-RBD**	2	3	5	0	16
**omicron-RBD**	15	14	16	16	0

Figure S9. Amino acid sequence alignment of SARS-CoV2 RBD variants. (a) Alignment of 223 consecutive amino acids residues from S protein affected by mutations: WT-RBD (Wuhan-Hu-1) Sino Biological Beijing,P.R.China (cod. 40592-V08H); alpha-RBD (B.1.1.7) (N501Y) Sino Biological (cod. 40592-V08H82); gamma-RBD (P.1/ P.1.1/ P.1.2) (K417T, E484K, N501Y) Sino Biological (cod. 40592-V08H86); delta-RBD (B.1.617.2) (L452R, T478K) Sino Biological (cod. 40592-V08H90); omicron-RBD (B.1.1.529) (G339D, S371L, S373P, S375F, K417N, N440K, G446S, S477N, T478K, E484A, Q493R, G496S, Q498R, N501Y, Y505H). Asterisk, colon and period symbols indicate the positions which have a single, fully conserved residue (*), or a conservation between groups of strongly similar properties (:), or a conservation between groups of weakly similar properties (.), respectively. (b) Number of amino acids by which the RBD protein sequences differ.

1.12 Supplementary Figure 10

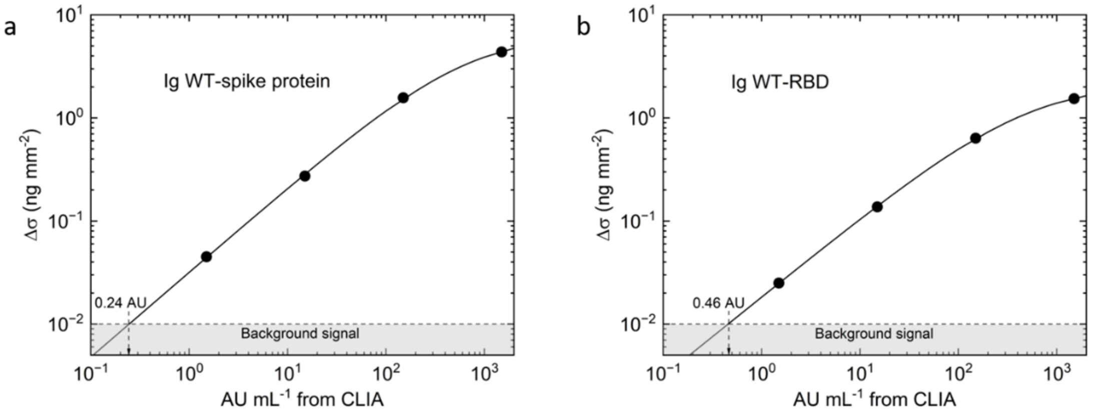

Figure S10. Calibration and limit of detection of the RPI antigen micro-array. Surface density of immunoglobulins binding on spots of (a) WT-spike protein and (b) WT-RBD measured by RPI for sample VX14 as a function of the amount of IgG antibodies quantified by Chemiluminescence Enzyme Immunoassays (CLIA kit DiaSorin LIAISON SARS-CoV-2 S1/S2 IgG) expressed as Arbitrary Units per milliliter (AU mL^{-1}). The black curves represent fits to the data point with the function $\Delta\sigma = \Delta\sigma_\infty/[1+(K_D/c)^p]$, where $\Delta\sigma_\infty$ is the asymptotic surface density that saturates the surface binding sites, K_D is the equilibrium dissociation constant, c is the IgG concentration measured by CLIA. The values of the parameters obtained from the fit are: $\Delta\sigma_\infty$= 6.6 ng mm^{-2} (a) and $\Delta\sigma_\infty$ = 2.2 ng mm^{-2} (b); K_D = 650 AU mL^{-1} (a) and K_D = 495 AU mL^{-1} (b); p = 0.82 (a) and p = 0.77 (b). The horizontal dashed lines represent the background signal obtained as the maximum non-specific binding response on SARS-CoV-2 antigen spots when plasma sample acquired before the Covid-19 pandemic is added in the measuring cartridge. The limit of detection is indicated by the arrows.

1.13 Supplementary Figure 11

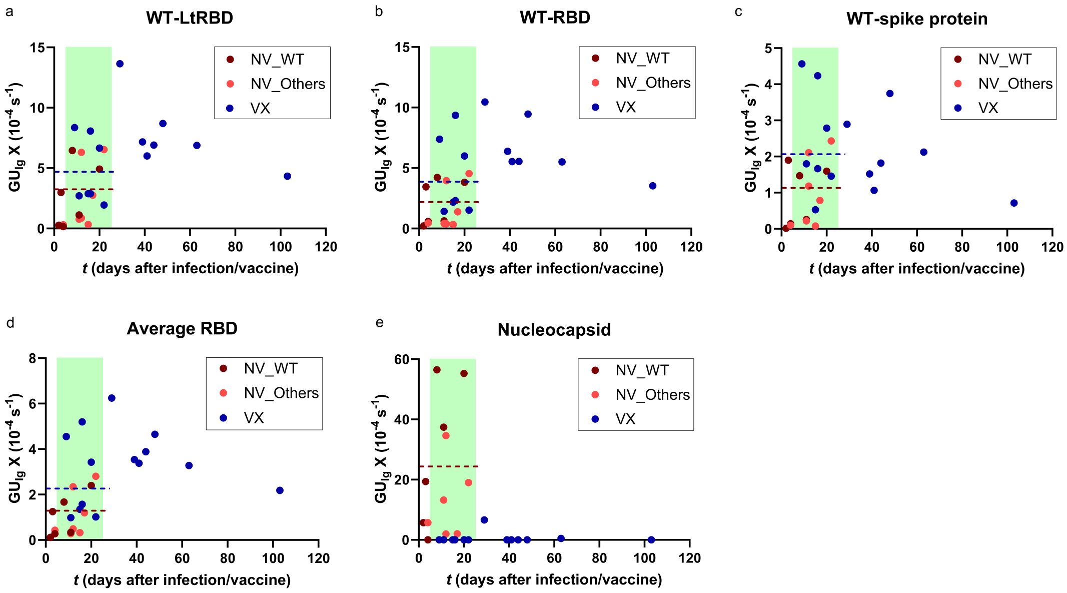

Figure S11. Dependence of Ig level on the time after 2nd vaccine dose or from the onset of symptoms. The reported values of GU_{Ig} correspond to antibodies binding on spots of WT-LtRBD (a), WT-RBD (b), WT-spike protein (c), all spots of RBD variants (d) and spots of nucleocapsid (e). The colour of the data points refers to Ig levels of vaccinated subjects as a function of days after the 2nd dose of vaccine (blue dots) or of unvaccinated subjects infected with WT variant (dark red) or other variants (light red) as a function of days from the onset of symptoms. The green band corresponds to the time window in which vaccinated and unvaccinated samples overlap. The dotted lines are the average values for vaccinated (blue) and all unvaccinated (red) subjects in the overlapping region. It is well known that serum immunoglobulin levels targeting SARS-CoV-2 progressively increase after a few days from vaccination or infection and then tend to decrease in weeks or months (Peterhoff et al. (2020), Gaebler et al. (2021)). The results are consistent with this behaviour, although the limited time range of the analysed samples does not allow estimating a significant time dependence of the measured parameters. Notably, despite the fact that the samples collected from vaccinated subjects have a larger range of time after the exposure to the virus antigens relative to convalescent subjects, the observations of larger Ig levels and smaller IgA levels for the vaccinated subjects are confirmed also comparing samples collected within similar time ranges.

1.14 Supplementary Figure 12

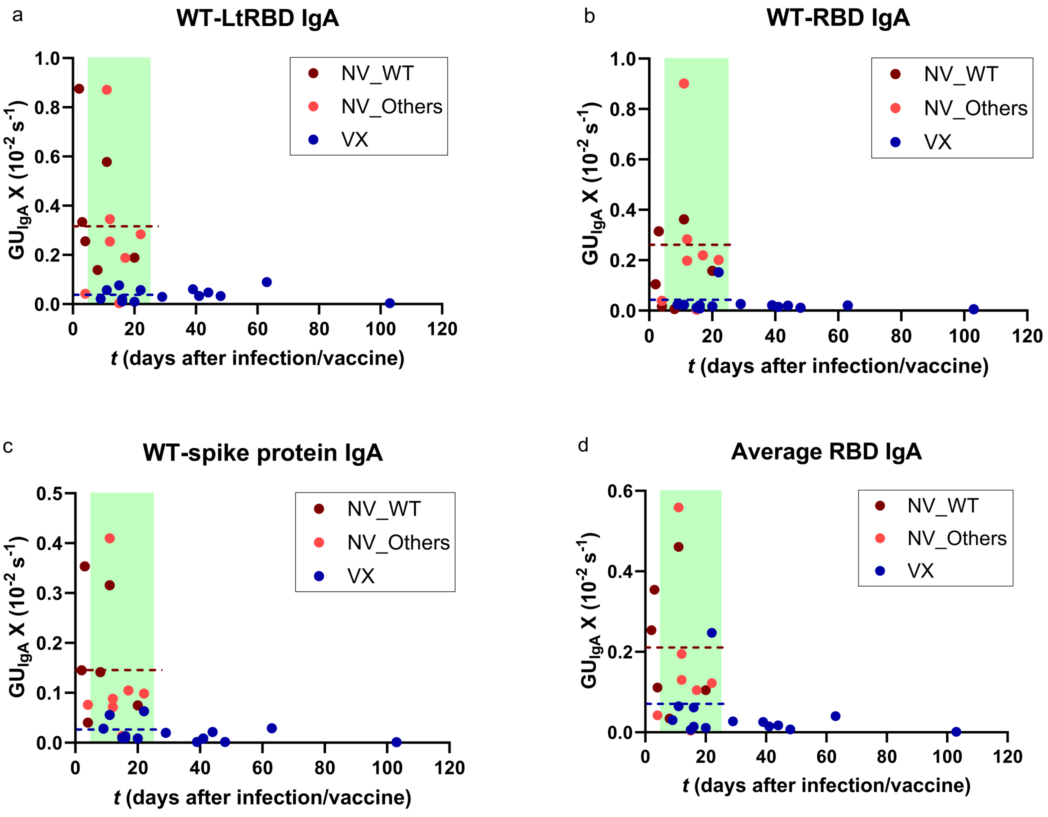

Figure S12. Dependence of IgA level on the time after 2nd vaccine dose or from the onset of symptoms. The reported values of GU_{IgA} correspond to antibodies binding on spots of WT-LtRBD (a), WT-RBD (b), WT-spike protein (c), and all spots of RBD variants (d). The colour of the data points refers to IgA levels of vaccinated subjects as a function of days after the 2nd dose of vaccine (blue dots) or of unvaccinated subjects infected with WT variant (dark red) or other variants (light red) as a function of days from the onset of symptoms. The green band corresponds to the time window in which vaccinated and unvaccinated samples overlap. The dotted lines are the average values for vaccinated (blue) and all unvaccinated (red) subjects in the overlapping region.

2 SUPPLEMENTARY NOTE

2.1 Supplementary Note S1

Principal component analysis (PCA) was performed on the whole data set of Ig and IgA data. PCA enabled determining linear orthogonal combinations of the parameters (in this case the Ig and IgA quantification), named PC1, PC2, PC3 etc. that maximally spread out the value among the 27 subjects, hence carrying the largest amount of information on the data. Accordingly, the variance between the value of PC1 computed on the set of subjects is the largest among any other combination of GU_{Ig} and GU_{IgA} values. PC1, PC2 and PC3 are obtained by multiplying the 15 GU_{Ig} and GU_{IgA} values by optimized arrays of coefficients that are reported in Figure 4c. PCA was made with a Python (v. 3.9.2) routine, using the machine learning library scikit-learn (v. 1.2.0). sklearn.decomposition.PCA() class performed a linear dimensionality reduction through Singular Value Decomposition (SVD). Multidimensional were rescaled to unit variance and centred around zero prior to processing using the .fit_transform(method) of the sklearn.preprocessing.StandarScaler() class.

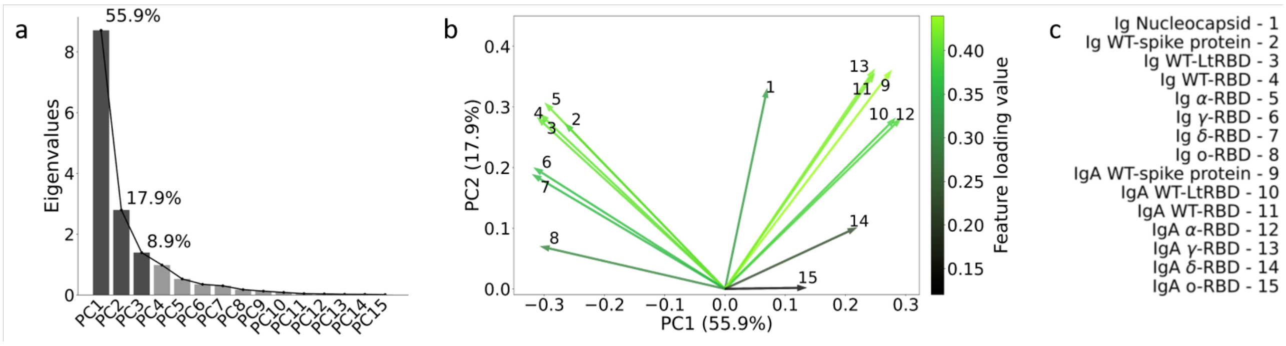

Figure S13. Principal component analysis of antibody fingerprints. (a) Scree plot of PCA, which visually presents the eigenvalues and their corresponding explained variance for the first three components. The eigenvalues represent the amount of variance captured by each component, with higher eigenvalues indicating components that explain more variability in the data. The explained variance (reported as percentage), shown alongside the eigenvalues, quantifies the proportion of total variance accounted for by each respective component. The scree plot aids in determining the significance and contribution of each component in summarizing the dataset's variability and assessing the optimal number of components to retain in the analysis. (b) The arrows centered in the origin represent the projection of each feature on the PC1-PC2 plane. The color of the arrows represent the loading value of each feature, according to the color bar. (c) Antibody type and antigen spot associated to each feature of panel b.

2.2 Supplementary Note S2

To demonstrate the feasibility of a pseudo-neutralization assay (Johnson et al. (2020)) based on RPI label-free micro-array, we analyzed a plasma sample of an unvaccinated convalescent subject. The infection occurred in the first months of the pandemic, hence it is expected to be caused by the WT virus variant, although the variant of infection was not determined by molecular test. We first measured the binding of immunoglobulins contained in this plasma sample to the RBD proteins from different variants. Then, after washing, instead of adding anti-IgA antibodies, we added in solution the extracellular domain of recombinant human angiotensin-converting enzyme 2 (ACE2) expressed in HEK293 cells (RayBiotech Life Inc., USA, cat. no. 230-30165) at a concentration of 35 nM and measured its binding to the RBD spots after the exposure to the blood sample. To quantify the inhibition of the binding of ACE2 to the surface antigens due to the binding of serum antibodies, we compared the ACE2 binding profile to a similar profile obtained from a reference sample of plasma acquired before the pandemic. Supplementary Figure S14 reports the binding of immunoglobulins (panels a-c) and of ACE2 (panels d-f) to the surface antigens. The Ig fingerprint (panel c) is consistent with an infection due to WT variant. Comparing the binding profiles of the sample from the convalescent subject (panel e) and that from the reference sample (panel d), we obtained a profile for ACE2 binding computed as the ration of the RGU for each variant for the two samples (panel f). The results show a clear correlation between the level of immunoglobulins binding on each RBD spot and the inhibition of ACE2 binding, suggesting a correlation between the measured Ig fingerprint and Ig neutralization capability. However, further work is required to rule out possible effects due to different affinity and kinetics of the Ig-antigen binding.

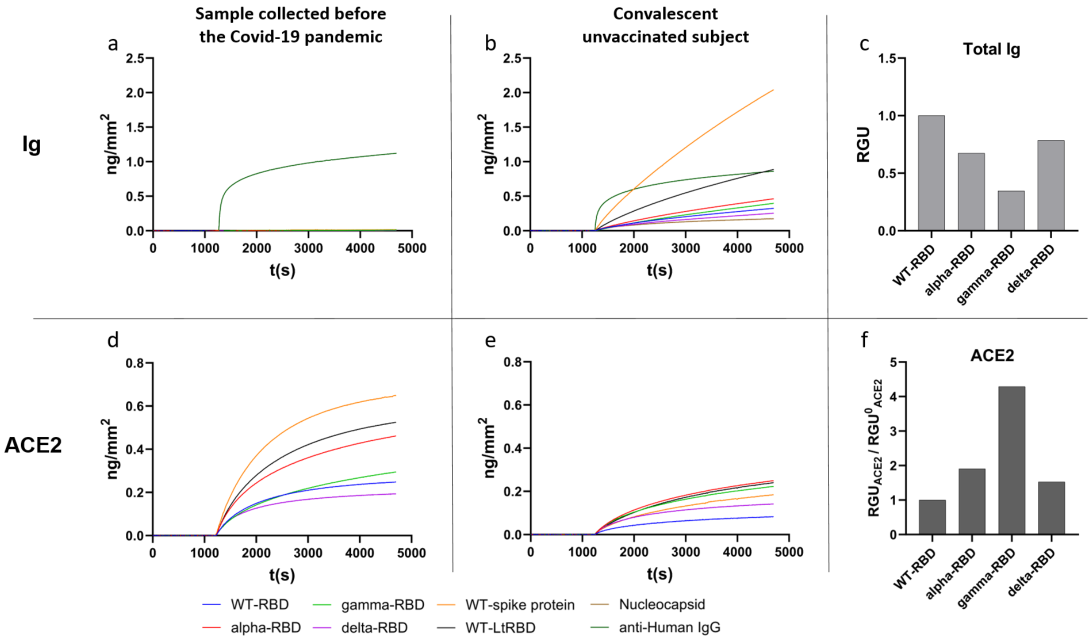

Figure S14. Demonstration of pseudo-neutralization assay by RPI label-free microarray. (a) Surface density measured upon addition of plasma sample collected before the Covid-19 pandemic. (b) Surface density measured upon addition of plasma sample of a convalescent unvaccinated subject infected in December 2020, when the prevalent strain in Italy was B.1.177 (WT-ES). (c) RGU obtained from the data of panel b as the ratio between the GU of each variant RBD and that of WT-RBD. (d) Surface density of ACE2 receptor binding the surface-immobilized antigen after the first step of the assay reported in panel a, performed with plasma sample collected before the Covid-19 pandemic. (e) Surface density of ACE2 receptor binding the surface-immobilized antigen after the first step of the assay reported in panel b, performed with plasma sample of a convalescent unvaccinated subject. (f) Ratio between the values of RGU obtained from the data of panel e and those obtained from the data of panel d.

REFERENCES

Gaebler, C., Wang, Z., Lorenzi, J. C. C., Muecksch, F., Finkin, S., Tokuyama, M., et al. (2021). Evolution of antibody immunity to SARS-CoV-2. *Nature* 591, 639–644. doi:10.1038/s41586-021-03207-w

Johnson, M., Wagstaffe, H. R., Gilmour, K. C., Mai, A. L., Lewis, J., Hunt, A., et al. (2020). Evaluation of a novel multiplexed assay for determining igg levels and functional activity to sars-cov-2. *Journal of Clinical Virology* 130, 104572. doi:10.1016/j.jcv.2020.104572

Peterhoff, D., Glück, V., Vogel, M., Schuster, P., Schütz, A., Neubert, P., et al. (2020). A highly specific and sensitive serological assay detects SARS-CoV-2 antibody levels in COVID-19 patients that correlate with neutralization. *Infection* 49, 75–82. doi:10.1007/s15010-020-01503-7
